# Important Ethical, Technical, and Epidemiological Considerations in an AI Tool Production (ETEPAI): Scoping Review

**DOI:** 10.2196/80340

**Published:** 2026-03-05

**Authors:** Boon How Chew, Kee Yuan Ngiam

**Affiliations:** 1 Department of Family Medicine, Faculty of Medicine and Health Sciences, Universiti Putra Malaysia, Serdang, Selangor, Malaysia; 2 Family Medicine Specialist Clinic, Hospital Sultan Abdul Aziz Shah, Universiti Putra Malaysia, Serdang, Selangor, 43400 UPM, Malaysia, 60397692538; 3 Department of Biomedical Informatics, Yong Loo Lin School of Medicine, National University of Singapore, Singapore, Singapore; 4 Division of General Surgery (Thyroid and Endocrine Surgery), Department of Surgery, National University of Singapore, Singapore, Singapore

**Keywords:** artificial intelligence, ethics, epidemiology, clinical decision support systems, practice guidelines

## Abstract

**Background:**

Artificial intelligence (AI) tools are being developed in a rapidly evolving technology. The convergence of ethical, technical, and research methods’ considerations is crucial for multidisciplinary teams aiming to produce effective AI tools. The success of these tools postdeployment hinges on the intricate interplay between the AI system’s development on its output through rigorous decision-making processes and stakeholders’ capacity to act on the AI’s recommendations.

**Objective:**

This paper synthesizes ethical, technical, and epidemiological considerations for all involved in artificial intelligence tool production (ETEPAI), based on established guidelines, checklists, and frameworks.

**Methods:**

Relevant guidelines, checklists, frameworks, and expert recommendations were systematically identified and synthesized into ETEPAI, an ethical, technical, and epidemiological framework for AI tool development in health care.

**Results:**

From 30 reviewed frameworks, ETEPAI integrates critical considerations across 4 stages (design, development, deployment, and postdeployment) and 3 domains (ethics, technical, and epidemiological), providing a compact yet comprehensive guide. It includes probing questions, key indicators, and common pitfalls to support high-quality, ethically sound, and clinically relevant AI tools. ETEPAI aligns with European Union trustworthiness standards and is supported by a research proposal template and supplementary references to aid implementation and adoption. We present probing questions and critical pointers across 4 stages from the design, development, deployment, and postdeployment, highlighting their relevance in health care settings. The designing stage aligns with epidemiologic research methodologies, while the development stage emphasizes transparent project execution. Deployment and postdeployment stages focus on real-world implementation. Additionally included are common pitfalls and challenges to emphasize the importance of due attention to the importance of ETEPAI considerations to avoid serious consequences.

**Conclusions:**

Applying ETEPAI ensures comprehensive, complete, compact, and crisp consideration from conception to execution, promoting high-quality, ethically sound, and clinically relevant AI tools. The brevity and conciseness of ETEPAI might be adequate for trained personnel and serve as clear signposts to unprepared stakeholders.

## Introduction

In the rapidly evolving landscape of artificial intelligence (AI) development, the convergence of ethical, technical, and research methods considerations is paramount for multidisciplinary teams aiming to achieve success in the production of AI tools [1]. The impact of AI tools after deployment depends on the intricate interplay between the AI system’s development, the decision-making based on its output, and the capacity of the stakeholders involved to take the necessary subsequent actions. Foreseeing, estimating, and designing the AI tools that meet all the challenges before deployment are essential to bridge the chasm between AI tools development and achievable benefits [2] with acceptable risks [3].

However, there is a proliferation of guidelines, checklists, assessments, frameworks, and recommendations [4,5] which could lead to confusion, inconsistency, and inefficiency in adherence and application [6]. Overwhelmed by the many similar yet different and lengthy referent materials, developers and stakeholders may overlook the most relevant and essential practices, rendering many good referent materials less effective, stalling improvement in the AI tools production process, or risking the perpetuation of the poor conduct and reporting of such studies [7]. These include a lack of data and code availability, an absence of or small human comparator groups, and a shortage of real-world clinical relevance, transparency, and inappropriate conclusions, causing overall high risk of bias [8], leading to more research waste [9-11]. To address these issues, there is a pressing need to integrate the various referent materials into a unified, thorough, concise, and clear approach across different study designs to inform about the best practices and ethical standards that accentuate the clinical relevance of AI tools and systems production that integrate into clinical workflow for sustainability throughout the whole developmental process.

For the purposes of this review, we use the term “artificial intelligence” as a broad umbrella to encompass the spectrum of machine learning (ML) models commonly used for clinical tasks, from traditional regression to deep learning. While the technical complexity of these algorithms varies, the fundamental principles of ethical oversight, rigorous epidemiological validation, and postdeployment governance required for safe clinical integration remain consistent.

## Methods

### Search Strategy and Selection Criteria

Searches for relevant recommendations were conducted through a multipronged search strategy combining systematic database searches with supplementary methods to capture a broad range of relevant recommendations. First, a systematic search was conducted in key academic databases, including PubMed, Google Scholar, and Semantic Scholar. The search strategy used a combination of keywords and Boolean operators to identify relevant literature. A representative search string was: ("artificial intelligence" OR "machine learning") AND ("guideline" OR "framework" OR "checklist" OR "recommendation" OR "best practice") AND ("healthcare" OR "clinical" OR "medicine"). Second, this initial search was supplemented by several methods. We conducted a targeted review of regulatory documents from prominent health and technology organizations, published policy, and gray literature. We also performed citation searching by manually reviewing the reference lists of highly relevant guidelines and systematic reviews to identify eligible papers. This process was further enriched through consultation with domain experts to ensure seminal works were not overlooked. Additionally, we used AI-powered academic search tools such as Elicit, SciSpace, and Perplexity to identify relevant preprints and newly published papers that may not have been fully indexed in traditional databases. Recommendations that were selected must be developed by groups of experts through a systematic and scientific process, provide explicit, actionable recommendations for the design, development, validation, or deployment of clinical AI tools, and must be published in English.

### Data Extraction and Synthesis

**Figure 1.**
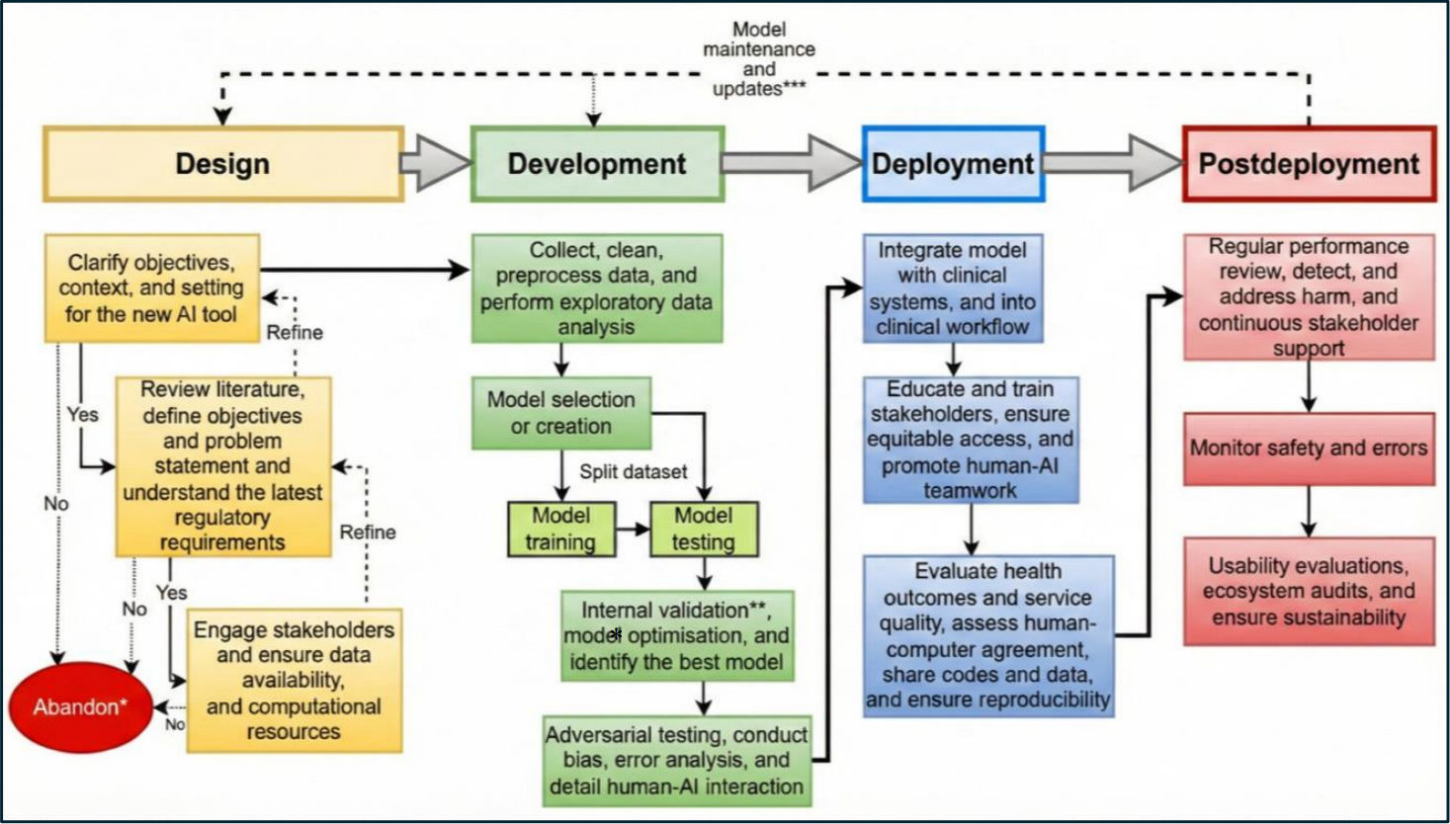
Key stages of AI tools from design to postdeployment. *No responses that lead to an abandonment include nonethical conduct, superior alternatives, infeasibility of whatever causes, nonacceptance by users, strategic or value misalignment to the institutions involved, financial viability, and economic factors to sustain. **May include external validation, decision curve analysis, (early) health technology assessments, and impact studies such as randomized controlled clinical trials. ***Model maintenance and updates may require a revisit from the beginning or to the tuning in the development stage. AI: artificial intelligence.

The included recommended guidelines [3,12-16], checklists [17-23] and the AIPA (Artificial Intelligence Prediction Algorithm) for medical sectors [24], the Stanford’s FURM (Fair, Useful, the Reliable Artificial Intelligence Model) assessment [2], the SUDO (pseudo-label discrepancy) framework [25], and the medical algorithmic audit framework [26], and the STANDING (Standards for Data Diversity, Inclusivity, and Generalisability) Recommendations [27] are summarized with the checklists of the referenced materials provided via links on the tables in the Multimedia Appendix 1 [3,12,13,16-21,24,26-51], and excluded guidelines were discussed further and described in Multimedia Appendix 2 [52-66]. We assimilated the referenced materials into important ethical, technical, and epidemiological considerations for all involved in artificial intelligence tool production (ETEPAI) as critical pointers according to 4 coherent stages of design, development, deployment, and postdeployment in 3 domains of ethics, technical, and epidemiological principles (Figure 1). While these 4 stages are analogous to a standard software development life cycle to ensure familiarity and ease of integration, the critical pointers within each stage are specifically tailored to the unique challenges of developing safe and effective AI tools for health care.

Important concepts and must-have indicators from the referenced materials are deemed sufficient and included in the list of items in ETEPAI as they represent well the rest of the indicators in their respective material. To prime users at the start of using ETEPAI are probing questions that aim to enhance readiness (Textbox 1). Added to these guides on best practices is a section on the common pitfalls and prevailing challenges to highlight and re-emphasize the importance of due consideration to every step in the AI tools development in order to avoid serious consequences. In this approach, ETEPAI is more inclusive and thorough compared to any of the referenced materials. ETEPAI considerations are comprehensive, complete, compact, and crisp to use to guide high-quality, ethically sound, and clinically relevant AI tools from idea conception to plan execution, and from promoting to monitoring. As a result, we also provided an AI research proposal template in Multimedia Appendix 3. Thus, ETEPAI serves as annotated content pages of books that signpost authors and users to the necessity of each step in the process but would require further reading in order to gain further understanding if this did not happen before. Similarly, readers and users of this ETEPAI are advised to refer to the original references for full explanation (Multimedia Appendix 1), and to other more elaborative literatures [67], on rigorous external model validation [16,68-70] and for clinical trial and economic evaluation [21,23,28], or for additional help in complete reporting guidelines [5,71], in organizational capabilities strengthening for AI adoption [72], in sociotechnical dimensions to consider when integrating AI tools in complex adaptive health care systems [73], and to identify technological solutions for achieving large-scale sustained adoption [74]. The scale and brevity of ETEPAI may be sufficient for trained personnel and serve as a sure sign to unmotivated stakeholders, as a standard evaluation and as materials to equip organizations to be AI-capable [72]. Further elaboration on its uses is given below.

ETEPAI’s product-centric approach could effectively align with European Union (EU)–defined trustworthiness standards [29,75] by emphasizing safety, robustness, and ethical considerations at every stage of the AI life cycle, from design through deployment. With its focus on rigorous processes, techniques, and verifiable methods, ETEPAI promotes comprehensive risk identification and mitigation strategies that address both product-level and system-level impacts on users and society. This focus directly supports compliance with the EU’s safety, health, and fundamental rights protections, ensuring AI systems are both reliable and ethically responsible. While ETEPAI aligns well with the principles of the EU AI Act, its fundamental focus on data privacy, responsible data handling, and accountability ensures its principles are compatible with and supportive of other major regulatory frameworks, such as HIPAA (Health Insurance Portability and Accountability Act) in the United States [76].

Textbox 1. **Probing questions in the 4 stages of artificial intelligence (AI) tools design, development, deployment, and postdeployment.**

Design

- What problem does this AI model seek to solve?
- Is the AI tool necessary and appropriate?
- How will the AI tool be used?
- Is the context in which the AI tool will be used appropriate?
- When and where will it be used or not used?
- Should a health care provider use the AI tool? Who else will use it?
- Will there be secondary (indirect) users of the AI tool?
- What are the main functions of the AI tool?
- What are the expected outcomes, potential secondary, and unexpected outcomes?
- How will the output of the model be used?
- How might this model impact patients, personnel, and the health care system?
- What would be the impact and consequences of the unexpected outcomes?
- What approaches would mitigate risks arising from the use of the AI tool?
- Given the cost and work capacity involved, what net benefits could be realized?
- Would the AI model-guided workflow be financially sustainable?

Development

- What are the available resources, data sources, and potential trade-offs for the AI system and technology?
- How should the objectives and functions of the AI tool be prioritized according to the available resources?
- Reuse an existing model or learn a new one?
- How to get the best and fairest model within the required time?

Deployment

- Is the deployment process the simplest possible for the acceptable iteration speed?
- How to get the outputs back into the clinical workflow?
- Are the validity and efficiency of the AI tool limited over time?
- How long can the results or the technology that supports the AI tool be used?

Postdeployment

- Does the AI model have the intended impact?
- How often should the AI tool or system be updated?
- Who is responsible for updating the AI system?
- Is governance in place to monitor the AI tool or system for its safety and errors, to audit the ecosystem within the components of larger societal systems?

## Results

### Critical Pointers and Domains

**Figure 2.**
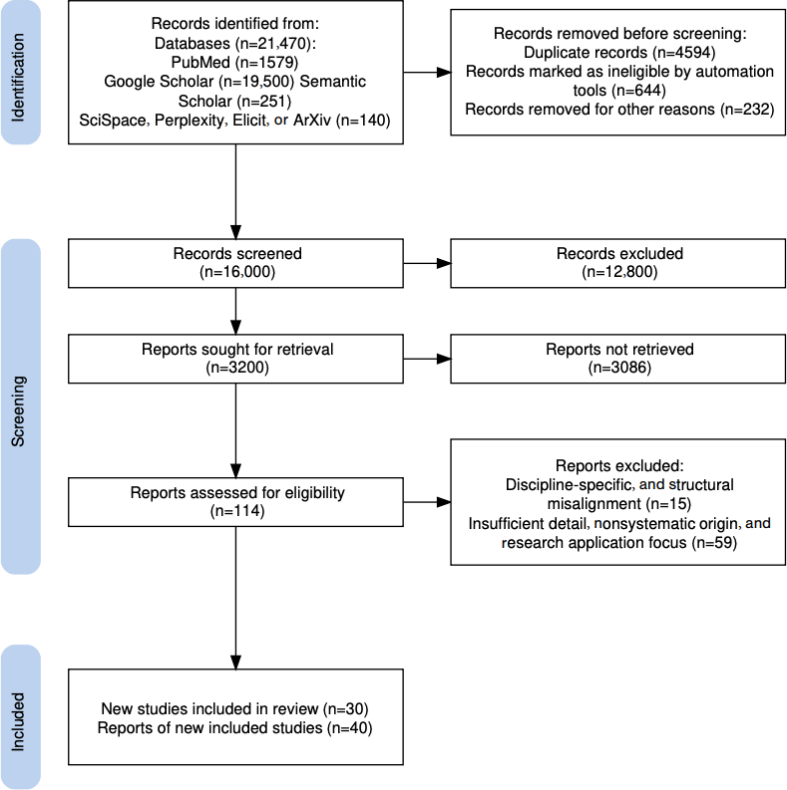
Flow diagram of sources screened and assessed for eligibility.

Figure 2 shows the flow diagram [77]. Figure 1 illustrates the key stages of AI tools from development to postdeployment. Textbox 1 lists probing questions in the 4 stages of design, development, deployment, and postdeployment stages according to their relevance and importance when thinking about having an AI tool in a health care setting. These would alert and engage users’ attention to the scopes and contexts of ETEPAI. Similarly, Table 1 provides critical pointers in AI tools production arranged in 3 domains of ethics, technical, and epidemiological principles under the same 4 stages. Although the probing questions and the critical pointers are grouped into different stages and domains, they are all to be considered when any AI tool is being conceived by an individual or discussed by a team of people, which should either result in a comprehensive research project proposal or abandonment if it is found to be unsound or a nonfeasible idea. Please see Checklist 1 for PRISMA-ScR (Preferred Reporting Items for Systematic Reviews and Meta-Analyses extension for Scoping Reviews) documentation.

**Table 1.** Critical pointers in 3 domains of ethics, technical, and epidemiological principles when considering an AI^a^ tool.

	Design	Development	Deployment	Postdeployment
Core concept	Clarify the objectives, context, and setting for the AI tool. Adopt standards and best practices to guide the whole design, development, and deployment processes to ensure compliance and interoperability of the AI technology with the health systems.	Conduct according to the proposed designs and approaches, which are made publicly available as a register on an open platform, a preprint, or a published journal paper.	Engage and educate multiple stakeholders for deployment and maintenance.	Evaluate the impact and improve performance of the deployed AI tool and system.
Ethical principles	Define relevant ethical issues through consultation, assess risk, and address biases. Prioritize safety in high-risk decisions and procedures. Promote transparency to assign responsibility, ensure trust in target users and the public, and protect patient rights. Address bias in the datasets for mitigation and not replication^b^. Safeguard the privacy of the datasets from any form of reidentification, and implement a dataset retention or removal plan. Responsibility and accountability among the model developer, IT^c^ staff at the deploying organization, and clinical staff are clearly defined. Limit the environmental impacts.	Preserve and enhance human autonomy in the use of the AI tool, where informed consent and the decision to refuse are allowed in the AI system. Maintain privacy and confidentiality in the collection and use of patient data, and uphold transparency of the AI system and its training data. Traceability and auditable AI methods^d^.	Reliability proven from testing in a similar setting. Nonmaleficence as harms and worst-case scenarios were considered. Ensure equitable access to the AI tool and related health care technologies and services.	Governance is in place to detect harm, redress plans, with a mechanism for humans to roll back, disengage, or deactivate the AI model. Institute regular challenges and reviews to monitor performance of the AI tool and system^e^.
Technical principles	Define problem statement and project scope, identify regulatory requirements, and policy framework^f^. Engage multiple stakeholders and understand contexts including geographical scope, users’ background, main languages, and digital skills^f^. Designs that are inclusive of all related stakeholders, effective preparation for source and learned code sharing. Data availability, quality^b^, and data procurement plans for data protection, high-quality annotation, and leakage prevention. Ensure sufficient computational resources^g^. Strive to achieve model interpretability and explainability^h^. Design the AI system with scalability and performance to handle growing data volumes and user demands^i^.	Conduct data collection as planned for all types and sources of data, data governance, and access^b^. Clean and preprocess the data to handle missing values, outliers, noise, and inconsistencies^b^. Perform exploratory data analysis to gain insights into the data and understand its characteristics^b^. Feature (variables) engineering to improve the performance of the model^j^. Model architecture and algorithm selection, and training that fit the project objectives^k^. Evaluation and validation to assess the performance, accuracy, and generalization capabilities of the model^l^. Hyperparameter tuning and optimization to fine-tune the model’s parameters and improve performance^m^. Design, data, and process audits^d^. Use adversarial attacks and red-teaming to identify worst-case behaviors^n^.	Clearly delineate responsibility for what to do, when, and how. Train stakeholders in why, how, and when to use the tool, including the main objectives, functions, and features, and differences among usage scenarios, when applicable^o^. Prepare the AI model for deployment in a production environment, integrate the model with the target system or application, and deploy it to production servers or cloud platforms. Promote effective human-AI teaming^h^. Implement mechanisms for model versioning, monitoring, and rollback to manage the deployment process effectively. Codes and data are shared with the community.	Documentation, knowledge transfer, and engage continuously with stakeholders and support users^p^. Establish a regular technical review to determine^e^ whether the AI tool is having the intended impact, is filling a gap in need, and is improving health care^q^. Maintenance, incorporating, verifying, and validating changes to the tool or system^e^. Ecosystem audit^r^, health economic evaluation, and/or full algorithmic audit^d^.
Epidemiological methods	Background: review existing relevant literature exploring AI models for the problem being addressed. Objective and problem: clearly state what the proposed AI tool aims to address with respect to the study setting, population, and outcome. Define the research question clearly^f^. Eligibility criteria for patients and features or input data, and rationale^s^. A fair distribution of the severity of disease and alternative diagnoses is required (spectrum bias). Time interval between index test and reference standard is within an appropriate interval^f^. Ground truth or referent standard: define the ground truth of interest, conditions, and outcome events, and rationale (if alternatives exist)^t^. Describe how it will be collected and encoded^u^. Define the test positivity cutoffs, distinguishing the prespecified from the exploratory. Source of data: describe how the dataset will be obtained and the study period^b^^,^^f^. Data abstraction, cleaning, and preparation to develop the final dataset^v^^,^^w^. Data splitting: specify how the data is to be divided into the training and testing cohorts^l^. Sample size estimation: provide rationale for sample size required for model development (eg, based on power calculation)^x^. Baseline model: describe the baseline model that will serve as a comparison for the AI model. Model description and evaluation: describe the software libraries to be investigated, the evaluation metrics to assess performance and calibration, transformation and optimization strategy^t^^,^^u^^,^^v^. Include clinical utility assessment, bias assessment, and error analysis (justify why not) beside the statistical methods to analyze the primary and secondary outcomes, subgroup analyses, and their rationale.	Model specification: specify the final panel of features included, ensure the independence between training and test sets^w^, and hyperparameters tuned^m^. The cohorts (training and test sets) are shown to be representative of real-world clinical settings (to be discussed if not), and missingness is addressed: reported, imputed, or corrected^y^. Clinical utility assessment: use appropriate metrics for the risk or benefit trade-offs at the specified decision threshold^z^. Validation or efficacy: nonclinical and clinical research for validation or to estimate the AI tool’s clinical effect in the routine clinical workflow, usability to those involved, and effects on clinical outcomes^z^. Bias assessment: compare evaluation metrics for the AI tool and reference standard, including stratification by patient- and task-specific subgroups, or subtyping^t^^,^^u^^,^^v^^,^^w^. Error analysis: analyze predictive errors to identify characteristics that are more prone to inaccurate predictions^x^^,^^t^^,^^u^^,^^v^. Determine if there are any surprise errors. Hyperparameter tuning: specify all model hyperparameters that are optimized, the search space for hyperparameter tuning, and evaluation metrics used to optimize parameters^m^. Specify whether there is human-AI interaction in the handling of the input data, and what level of expertise is required for users^h^. Explain the procedure for how the AI intervention’s output will contribute to decision-making or other elements of clinical practice^h^.	Model explanation and interpretability to improve acceptability, uptake, and sustainability^h^. Adoption: implement the AI system within the intended clinical workflow or care pathway^e^. Effectiveness: evaluate all patients’ health outcomes and service quality indicators, before and after deployment^aa^. Human-computer agreement: evaluate and report any instances of and reasons for user variation from the AI system’s recommendations, and if applicable, users changing their mind based on the AI system’s recommendations^aa^. Reproducibility: share the data, source code, or release an application that runs the code. A data dictionary involves providing descriptions of all features and ground truth.	Safety and errors: monitor and report any risks to patient safety or instances of harm. Provide a description of how significant errors or malfunctions were defined and identified^ab^ . Maintenance or sustainability: conduct usability evaluation, according to recognized standards or frameworks, the user learning curves evaluation, stakeholder and patients’ acceptability of the AI tools or systems. Support for the intended use of the AI system in clinical settings.

a AI: artificial intelligence.

b Ensure data adequacy by using representative, diverse, and sufficient data, properly labeled and curated to reduce bias. When using pre-existing cohorts for case-control sampling, make necessary sampling adjustments (eg, reweighting) to ensure correct calibration. Maintain process transparency by documenting the entire data pipeline, including collection methods, dataset purpose, and handling of missing or complex data. Finally, detail all known biases, disparate outcomes, or data shifts; describe mitigation procedures; and transparently report on any formal bias and fairness assessments (STANDING Together 2023 [Standards for Data Diversity, Inclusivity, and Generalisability Together 2023] [27], AIPA [Artificial Intelligence Prediction Algorithm] [24], APPRAISE-AI [Tool for Adapting Practice Parameters for Reporting Artificial Intelligence Studies in Healthcare] [17], CODE-EHR [Clinical Outcomes in Digital Enterprise - Electronic Health Records] [19], TRIPOD-AI [Transparent Reporting of a Multivariable Prediction Model for Individual Prognosis or Diagnosis - Artificial Intelligence] [16], ALTAI [Assessment List for Trustworthy Artificial Intelligence] [13,30], the medical algorithmic audit [26], and UN Resolution on AI [United Nations Resolution on Artificial Intelligence] 2024 [31]).

c IT: information technology.

d Artificial intelligence auditing is a critical mechanism for accountability in system decisions, with several key approaches: data audits evaluate training data; process audits scrutinize development documentation; and ecosystem audits assess human-AI interactions. This practice of algorithmovigilance, best conducted with all stakeholders, proactively identifies vulnerabilities to mitigate risk, guide critical thinking on system acceptability, and inform future model improvements. Audits should be complemented by related impact assessments and health economic evaluations to estimate cost-effectiveness (three key questions [78], the medical algorithmic audit [26], ALTAI [Assessment List for Trustworthy Artificial Intelligence] [13,30], FUTURE-AI [Fairness, Universality, Traceability, Usability, Robustness, and Explainability - Artificial Intelligence] [32], UNESCO Recommendation on the Ethics of Artificial Intelligence 2022 [United Nations Educational, Scientific and Cultural Organization Recommendation on the Ethics of Artificial Intelligence 2022] [33], and CHEERS-AI [Consolidated Health Economic Evaluation Reporting Standards for Interventions That Use Artificial Intelligence] [28,34]).

e Establish a maintenance plan to regularly monitor model performance, data quality, and user feedback. This is critical for addressing performance degradation caused by factors such as data distribution shift (concept drift) or inconsistencies from new devices, and for guiding how to iterate on the artificial intelligence system with necessary updates based on evolving requirements (APPRAISE-AI [Tool for Adapting Practice Parameters for Reporting Artificial Intelligence Studies in Healthcare] [17], TRIPOD-AI [Transparent Reporting of a Multivariable Prediction Model for Individual Prognosis or Diagnosis - Artificial Intelligence] [16], FUTURE-AI [Fairness, Universality, Traceability, Usability, Robustness, and Explainability - Artificial Intelligence] [32], ALTAI [Assessment List for Trustworthy Artificial Intelligence] [13,30], UNESCO Recommendation on the Ethics of Artificial Intelligence 2022 [United Nations Educational, Scientific and Cultural Organization Recommendation on the Ethics of Artificial Intelligence 2022] [33], and STANDING Together 2023 [Standards for Data Diversity, Inclusivity, and Generalisability Together 2023] [27]).

f Clearly define the artificial intelligence application’s context by specifying its medical indication, target population, intended end user (eg, specialist or patient), and the health care process it aims to improve with its expected benefits. The application’s timing of use within the clinical workflow and its type (eg, diagnostic, prognostic, and monitoring), including any prediction horizon, must also be defined. Crucially, ensure active stakeholder engagement to align with user needs, build trust, and ensure usability (AIPA [Artificial Intelligence Prediction Algorithm] [24], APPRAISE-AI [Tool for Adapting Practice Parameters for Reporting Artificial Intelligence Studies in Healthcare] [17], and CODE-EHR [Clinical Outcomes in Digital Enterprise - Electronic Health Records] [19]).

g Evaluate the computational resources required for training and deploying the artificial intelligence model. Consider factors such as the complexity of the model, the size of the dataset, and the computational power needed for training, inference, and scaling (ALTAI [Assessment List for Trustworthy Artificial Intelligence] [13,30], APPRAISE-AI [Tool for Adapting Practice Parameters for Reporting Artificial Intelligence Studies in Healthcare] [17], TRIPOD-AI [Transparent Reporting of a Multivariable Prediction Model for Individual Prognosis or Diagnosis - Artificial Intelligence] [16], and UNESCO Recommendation on the Ethics of Artificial Intelligence 2022 [United Nations Educational, Scientific and Cultural Organization Recommendation on the Ethics of Artificial Intelligence 2022] [33]).

h Consider the importance of model interpretability and explainability to enhance transparency into how the artificial intelligence model makes decisions. Various methods can provide insights, such as SHAP (Shapley Additive Explanations) or LIME (Local Interpretable Model-Agnostic Explanations) for local and global feature importance, partial dependence plots to illustrate feature-outcome relationships, or saliency maps to highlight influential areas in images (AIPA [Artificial Intelligence Prediction Algorithm] [24], ALTAI [Assessment List for Trustworthy Artificial Intelligence] [13,30], APPRAISE-AI [Tool for Adapting Practice Parameters for Reporting Artificial Intelligence Studies in Healthcare] [17], TRIPOD-AI [Transparent Reporting of a Multivariable Prediction Model for Individual Prognosis or Diagnosis - Artificial Intelligence] [16], FUTURE-AI [Fairness, Universality, Traceability, Usability, Robustness, and Explainability - Artificial Intelligence] [32], The medical algorithmic audit [26], and UNESCO Recommendation on the Ethics of Artificial Intelligence 2022 [United Nations Educational, Scientific and Cultural Organization Recommendation on the Ethics of Artificial Intelligence 2022] [33]).

i Design the artificial intelligence system with scalability and performance in mind to handle growing data volumes and user demands. Consider distributed computing, parallel processing, and optimization techniques to improve efficiency and scalability (ALTAI [Assessment List for Trustworthy Artificial Intelligence] [13,30] and UN Resolution on AI 2024 [United Nations Resolution on Artificial Intelligence] [31]).

j Apply feature engineering techniques (eg, dimensionality reduction, scaling, and transformation) to improve model performance, and meticulously document all analytical and modeling procedures in sufficient detail to allow a third party to accurately reproduce the results (AIPA [Artificial Intelligence Prediction Algorithm] [24], APPRAISE-AI [Tool for Adapting Practice Parameters for Reporting Artificial Intelligence Studies in Healthcare] [17], TRIPOD-AI [Transparent Reporting of a Multivariable Prediction Model for Individual Prognosis or Diagnosis - Artificial Intelligence] [16], and FUTURE-AI [Fairness, Universality, Traceability, Usability, Robustness, and Explainability - Artificial Intelligence] [32]).

k Choose appropriate algorithms (eg, supervised and deep learning) based on project objectives, split the data into training, validation, and test sets, and clearly document all modeling techniques and stages from model selection to parameter tuning and calibration or recalibration to ensure reproducibility (AIPA [Artificial Intelligence Prediction Algorithm] [24], APPRAISE-AI [Tool for Adapting Practice Parameters for Reporting Artificial Intelligence Studies in Healthcare] [17], TRIPOD-AI [Transparent Reporting of a Multivariable Prediction Model for Individual Prognosis or Diagnosis - Artificial Intelligence] [16], and the medical algorithmic audit [26]).

l Validate the model against predefined criteria using appropriate internal validation methods (eg, bootstrapping and cross-validation), and assess its performance using a comprehensive suite of performance metrics, such as those for accuracy and precision, quantitatively and qualitatively [79] (AIPA [Artificial Intelligence Prediction Algorithm] [24], APPRAISE-AI [Tool for Adapting Practice Parameters for Reporting Artificial Intelligence Studies in Healthcare] [17], and TRIPOD-AI [Transparent Reporting of a Multivariable Prediction Model for Individual Prognosis or Diagnosis - Artificial Intelligence] [16]).

m Explore techniques such as grid search, random search, or Bayesian optimization to search for optimal hyperparameters efficiently (APPRAISE-AI [Tool for Adapting Practice Parameters for Reporting Artificial Intelligence Studies in Healthcare] [17] and TRIPOD-AI [Transparent Reporting of a Multivariable Prediction Model for Individual Prognosis or Diagnosis - Artificial Intelligence] [16]).

n Before real-world deployment, conduct adversarial attacks and red-teaming exercises where teams actively try to exploit vulnerabilities to proactively identify worst-case behaviors, potential for malicious use, and unexpected failures that fixed benchmarks might miss (APPRAISE-AI [Tool for Adapting Practice Parameters for Reporting Artificial Intelligence Studies in Healthcare] [17], TRIPOD-AI [Transparent Reporting of a Multivariable Prediction Model for Individual Prognosis or Diagnosis - Artificial Intelligence] [16], FUTURE-AI [Fairness, Universality, Traceability, Usability, Robustness, and Explainability - Artificial Intelligence] [32], ALTAI [Assessment List for Trustworthy Artificial Intelligence] [13,30], the medical algorithmic audit [26], and UNESCO Recommendation on the Ethics of Artificial Intelligence 2022 [United Nations Educational, Scientific and Cultural Organization Recommendation on the Ethics of Artificial Intelligence 2022] [33]).

o Ensure transparency and usability by providing a comprehensive model card. This should detail the model’s purpose, intended use, performance metrics, and methodologies, with information tailored to the specific needs of different end users, such as providing implementation details for clinicians and clear explanations of impact for patients (AIPA [Artificial Intelligence Prediction Algorithm] [24], ALTAI [Assessment List for Trustworthy Artificial Intelligence] [13,30], and UNESCO Recommendation on the Ethics of Artificial Intelligence 2022 [United Nations Educational, Scientific and Cultural Organization Recommendation on the Ethics of Artificial Intelligence 2022] [33]).

p For high-stakes tools on data without ground-truth labels, advanced techniques such as the SUDO (pseudo-label discrepancy) framework can identify unreliable predictions and bias. This should be part of a comprehensive documentation of the entire development process, with training materials provided to ensure effective knowledge transfer to stakeholders (APPRAISE-AI [Tool for Adapting Practice Parameters for Reporting Artificial Intelligence Studies in Healthcare] [17], TRIPOD-AI [Transparent Reporting of a Multivariable Prediction Model for Individual Prognosis Or Diagnosis - Artificial Intelligence] [16], FUTURE-AI [Fairness, Universality, Traceability, Usability, Robustness and Explainability - Artificial Intelligence] [32], ALTAI [Assessment List for Trustworthy Artificial Intelligence] [13,30], and UNESCO Recommendation on the Ethics of Artificial Intelligence 2022 [United Nations Educational, Scientific and Cultural Organization Recommendation on the Ethics of Artificial Intelligence 2022] [33]).

q Conduct thorough testing (eg, unit, integration, and system) to verify functionality and detect performance shifts caused by real-world dataset shifts (eg, population and prevalence). Pay close attention to performance gaps in specific subgroups (hidden stratification) and systematic algorithmic errors or failure modes [26] (APPRAISE-AI [Tool for Adapting Practice Parameters for Reporting Artificial Intelligence Studies in Healthcare] [17], TRIPOD-AI [Transparent Reporting of a Multivariable Prediction Model for Individual Prognosis or Diagnosis - Artificial Intelligence] [16], FUTURE-AI [Fairness, Universality, Traceability, Usability, Robustness, and Explainability - Artificial Intelligence [32]], ALTAI [Assessment List for Trustworthy Artificial Intelligence] [13,30], and the medical algorithmic audit [26]).

r Postdeployment analysis allows AI systems to be studied as components of larger societal systems; monitoring real-world usage, including emergent threats such as jailbreaks or deepfakes, is crucial for shaping scientific research on mitigating harms (ALTAI [Assessment List for Trustworthy Artificial Intelligence] [13,30], the medical algorithmic audit [26], and UNESCO Recommendation on the Ethics of Artificial Intelligence 2022 [United Nations Educational, Scientific and Cultural Organization Recommendation on the Ethics of Artificial Intelligence 2022] [33]).

s Inappropriate participant inclusion or exclusion criteria can harm generalizability, as the training data must be representative of the target population. Artificial intelligence systems perform well on in-distribution data (interpolation) but poorly on out-of-distribution data that requires extrapolation (STANDING Together 2023 [Standards for Data Diversity, Inclusivity, and Generalisability Together 2023] [27], TRIPOD-AI [Transparent Reporting of a Multivariable Prediction Model for Individual Prognosis or Diagnosis - Artificial Intelligence] [16], ALTAI [Assessment List for Trustworthy Artificial Intelligence] [13,30], and the medical algorithmic audit [26]).

t Bias can arise from flawed methods of outcome determination, such as using suboptimal or inconsistently applied criteria, incorrect timing, or knowledge of predictors influencing the assessment. A key issue is incorporation bias, where a predictor is part of the outcome definition, leading to overly optimistic performance estimates (APPRAISE-AI [Tool for Adapting Practice Parameters for Reporting Artificial Intelligence Studies in Healthcare] [17], CODE-EHR [Clinical Outcomes in Digital Enterprise - Electronic Health Records] [19], TRIPOD-AI [Transparent Reporting of a Multivariable Prediction Model for Individual Prognosis or Diagnosis - Artificial Intelligence] [16], and the medical algorithmic audit [26]).

u Bias can also be introduced by flawed predictor definition and measurement, such as when predictors are defined inconsistently across participants or when knowledge of the outcome influences their assessment (APPRAISE-AI [Tool for Adapting Practice Parameters for Reporting Artificial Intelligence Studies in Healthcare] [17], TRIPOD-AI [Transparent Reporting of a Multivariable Prediction Model for Individual Prognosis or Diagnosis - Artificial Intelligence] [16], and STANDING Together 2023 [Standards for Data Diversity, Inclusivity, and Generalisability Together 2023] [27]).

v For model development, ensure sufficient sample size, typically an events per variable of ≥20, and often >200 for artificial intelligence models to minimize overfitting and avoid selecting predictors based on univariable analysis. For model validation, a minimum of 100 participants with the outcome is recommended, and unlike for development, a priori sample size calculations are generally feasible (TRIPOD-AI [Transparent Reporting of a Multivariable Prediction Model for Individual Prognosis or Diagnosis - Artificial Intelligence] [16]).

w Select predictors based on existing knowledge and clinical credibility, not univariable analysis; retain important predictors regardless of statistical significance. Handle continuous predictors appropriately: avoid dichotomization, which loses information and risks bias, and instead model them continuously while examining for nonlinearity (eg, using fractional polynomials or splines; TRIPOD-AI [Transparent Reporting of a Multivariable Prediction Model for Individual Prognosis or Diagnosis - Artificial Intelligence] [16] and STANDING Together 2023 [Standards for Data Diversity, Inclusivity, and Generalisability Together 2023] [27]).

x While larger datasets are generally better, especially for complex models or class imbalance, this must be balanced with ethical data minimization. For models where a priori sample size formulas are unavailable, use a posteriori methods such as learning curves to assess data sufficiency and minimize overfitting (AIPA [Artificial Intelligence Prediction Algorithm] [24] and ALTAI [Assessment List for Trustworthy Artificial Intelligence] [13,30]).

y For a robust analysis, include all participants, handling missing data with appropriate methods such as multiple imputation. Evaluate both calibration and discrimination, and use internal validation techniques (eg, bootstrapping and cross-validation) to adjust performance estimates for model optimism (TRIPOD-AI [Transparent Reporting of a Multivariable Prediction Model for Individual Prognosis or Diagnosis - Artificial Intelligence] [16], APPRAISE-AI [Tool for Adapting Practice Parameters for Reporting Artificial Intelligence Studies in Healthcare] [17], STANDING Together 2023 [Standards for Data Diversity, Inclusivity, and Generalisability Together 2023] [27], FUTURE-AI [Fairness, Universality, Traceability, Usability, Robustness, and Explainability - Artificial Intelligence] [32], and the medical algorithmic audit [26]).

z Evaluate and document the model’s expected benefits using methods such as decision curve analysis, which assesses net benefit on decision-making, or a more comprehensive early health technology assessment to evaluate medical, economic, and social implications (AIPA [Artificial Intelligence Prediction Algorithm] [24], TRIPOD-AI [Transparent Reporting of a Multivariable Prediction Model for Individual Prognosis or Diagnosis - Artificial Intelligence] [16], and APPRAISE-AI [Tool for Adapting Practice Parameters for Reporting Artificial Intelligence Studies in Healthcare] [17]).

aa Evaluate potential outcomes by aligning with the quadruple aim (patient experience, cost, population health, and provider perception), considering a range of process, health, and societal impacts. A comparative study is essential to assess the tool’s added benefit, ideally a randomized comparative design, though alternatives such as controlled before and after studies may be used when randomization is not feasible (AIPA [Artificial Intelligence Prediction Algorithm] [24], ALTAI [Assessment List for Trustworthy Artificial Intelligence] [13,30], UNESCO Recommendation on the Ethics of Artificial Intelligence 2022 [United Nations Educational, Scientific and Cultural Organization Recommendation on the Ethics of Artificial Intelligence 2022] [33], and TRIPOD-AI [Transparent Reporting of a Multivariable Prediction Model for Individual Prognosis ﻿r Diagnosis - Artificial Intelligence] [16]).

ab For effective implementation, continuous monitoring is essential. This includes tracking performance and errors (eg, miscalibrations, false positives or negatives, and technical failures) and assessing fairness by analyzing outcome disparities across different populations to detect and mitigate bias (AIPA [Artificial Intelligence Prediction Algorithm] [24], ALTAI [Assessment List for Trustworthy Artificial Intelligence] [13,30], and UNESCO Recommendation on the Ethics of Artificial Intelligence 2022 [United Nations Educational, Scientific and Cultural Organization Recommendation on the Ethics of Artificial Intelligence 2022] [33]).

### ETEPAI Tool Production and Use

The designing stage is aligned with the epidemiologic research methodologies comprising the theoretical design, data collection design, and statistical analysis design [80,81]. The theoretical design involves identifying the real need for the AI tool, and defining the research question by having a clear target population, the exposure (or independent variable) of interest or interventional program, context or control, and the outcome measured at specified time point and setting (the PE/ICOTS [Population or Participants, Exposure/Intervention, Comparator, Outcomes, Timing, and Setting] acronym) [35,80]. The data collection is usually on already available datasets to be further selected and cleansed according to eligibility criteria. Prospective sampling is instructed by some guidelines. The statistical analysis includes the AI-specific models’ development or selection, evaluation, and validation procedures, estimating the best-performing AI-based prediction model. This is often summarized in terms of calibration and discrimination, without overoptimism, and with internal (cross-validation), external validation, decision curve analysis, (early) health technology assessments, and impact studies (such as via randomized clinical trials) [1]. The development stage is the conduct of the project according to the published and openly available project or research proposal. This allows public scrutiny of the project conduct from the proposed designs. Therefore, justification as a clear explanation must be given for every deviation in the actual conduct of the project from the proposal. The deployment and postdeployment stages are similar to the implementation research [82,83], where the tested AI tools are used in the routine practice. Deployment cost and organizational effort required to integrate the AI systems into a clinical workflow should be estimated to facilitate sustainability [78]. Most pointers in Table 1 have explanatory footnotes with further explanations in the Multimedia Appendix 4, and informative referenced materials given in parentheses. While the ethics domain has its referenced materials in Table S1 in Multimedia Appendix 1. This keeps the table within a readable limit and with adequate explanatory reference information. The explanatory footnotes are taken from the reference materials that are combined for maximal practical and educational clarity.

### Common Pitfalls and Prevailing Challenges

Afterthoughts and neglecting the aforementioned crucial considerations for AI tools development would inevitably cause serious problems and costly consequences. These pitfalls include inadequate data quality, biased algorithms, and misaligned AI capabilities that often emerge despite rigorous planning [84]. To re-emphasize the importance of due consideration of ETEPAI at every step throughout the development process to mitigate these risks [84], this section shares some potential pitfalls and appropriate preventive and corrective measures (Table 2) that can lead to models that perform effectively in real-world settings. These notable pitfalls and veracious challenges during AI tools development may vary according to different tools and settings. Further guides on best practices to remedy pitfalls, challenges, errors, and biases are available elsewhere [85,86].

**Table 2.** Potential pitfalls and possible best practices that could prevent and correct them. The content of this table is summarized from extracted materials in the study by Aliferis et al [86].

Number	Pitfalls	Best practices and measures
1	Using hard-to-reproduce, nonstandardized data input steps. Using nonrepresentative datasets or unusual populations and making wrong inferences.	Use high-quality and representative datasets and appropriate populations, and make claims based on appropriate datasets. Use dense time series data, leverage population models, and address abrupt distribution shifts in patient models.
2	Using normalization or data transforms requiring the entire sample, affecting test independence.	Use normalization or data transforms that do not require the entire sample (or confine such within discovery/training and validation datasets independently).
3	Not conducting power sample analysis or understanding sample size effects on modeling.	Perform reanalysis with improved protocols and domain knowledge.
4	Models are designed with excessive complexity relative to the data and sample size.	Manage model complexity using regularization, dimensionality reduction, feature selection, Bayesian methods, and model selection. Explore all relevant learning method families for better predictivity. Conduct power sample analysis and characterize sample size effects on modeling.
5	Failing to coordinate analysis across teams and datasets using unbiased, collaborative protocols.	Coordinate analysis across teams using unbiased protocols.
6	Not correcting for multiple statistical hypothesis tests.	Correct for multiple statistical hypotheses’ tests. Anticipate and incorporate multiple modeling stages to avoid overfitting.
7	Using biased estimators or introducing bias into otherwise unbiased ones.	Systematically explore the hyperparameter space.
8	Allowing uncontrolled iterative modeling, leading to analysis creep^a^.	Conduct iterative or sequential modeling using unbiased protocols. Follow theoretical and empirical specifications of reference methods.
9	Inappropriately modeling individual patients and overinterpreting generalizability.	Combine individual patient modeling with population modeling where possible. Use dense time series data, leverage population models, and address abrupt distribution shifts in patient models.
10	Not examining the stability of models and parameters, nor investigating unstable findings.	Examine the stability of models and parameters, and investigate unstable findings.
11	Allowing models to learn incorrect patterns due to spurious co-occurrence or uncontrolled structural relations.	Prevent models from learning incorrect patterns through proper control and domain knowledge.
12	Controlling only some factors contributing to overconfidence.	Control all relevant factors contributing to overconfidence via nested model selection.
13	Ignoring the statistical uncertainty of strong performance estimates.	Deploy and explore all relevant data preparation steps for the domain and task.
14	Insufficient evaluation of scalability and generalizability for bespoke models.	Use label reshuffling and independent dataset validation cautiously.
15	Reporting only the strongest models or results.	Fully report all procedures used to obtain models for independent verification. Inform analyses with methods literature to explore both best-known and novel methods.

a Analysis creep is the gradual introduction of biases or distortions into a model due to uncontrolled or repeated adjustments, often resulting in overfitting or overconfidence in the results.

### Common Pitfalls to Caution

In the rapidly evolving field of AI, developers and data scientists face a myriad of challenges that can significantly impact the effectiveness and reliability of their models. While much emphasis is placed on the theoretical foundations and potential applications of AI, it is equally important to be aware of the common pitfalls that can undermine these efforts. This section highlights several notable pitfalls that practitioners must navigate to ensure robust and accurate outcomes, from data preparation to model selection and beyond.

One major challenge in AI development is the narrow selection of models and methods [87]. A common pitfall is failing to explore the appropriate model family during model selection, such as using only linear regression models when the data-generating function is nonlinear and discontinuous [88]. Additionally, data scientists or vendors often have strong preferences for a limited set of methods or technologies, even when they are not the most suitable for the task [89]. This narrow approach can significantly hinder model performance and limit the potential of AI applications.

Another common issue is the tendency to rely on single-stage modeling attempts without refinement. Initial models typically require iterative enhancements, as first attempts rarely meet performance goals in complex problems. A single-stage approach lacks the iterative understanding necessary to optimize model interaction with data [90]. Furthermore, ignoring established best methods or misapplying robust methods outside of their intended use can result in underperforming models [91]. Adhering to proven methods and following established guidelines are critical for maintaining model integrity and achieving reliable outcomes.

Insufficient exploration of hyperparameters and data preparation is also a critical issue. Even when the right model families are chosen, inadequate tuning of hyperparameters can lead to suboptimal outcomes [92]. Similarly, improper data preparation, such as neglecting feature construction, selection, normalization, or discretization, can greatly impair a model’s effectiveness [93,94]. Thorough attention to both hyperparameter optimization and data preparation is essential for achieving robust model performance.

Data contamination and bias are additional challenges that can compromise model accuracy. When data processing steps such as normalization or discretization are conducted across both training and test sets, it can introduce bias and lead to inflated performance metrics [95]. This issue is exacerbated when nonrepresentative datasets or unusual populations are used, leading to models that do not generalize well. In some cases, models may produce misleading results by focusing on correlated factors rather than underlying causes, which can result in ineffective or incorrect conclusions.

Overconfidence in models, particularly in the context of overfitting and underfitting, poses a significant risk. Models may appear effective during development but fail in real-world applications due to high-dimensional data, small sample sizes, or overly complex learners [87]. This issue is closely related to the challenge of power-sample calculation in AI, which is more complex than in traditional statistical analysis. Learning curves, which describe the generalization error as a function of sample size, are often unknown, making it difficult to determine the required sample size for reliable model performance.

Addressing common pitfalls is essential for the effective development of AI models, but it is equally important to navigate the more complex challenges that can emerge. By adopting a balanced approach that includes both careful model selection and iterative refinement while also considering algorithmic transparency, regulatory impacts, and complete process reporting, developers can better ensure the reliability and ethical deployment of AI systems. These considerations are crucial for fostering innovation without compromising on responsibility, as AI continues to play an increasingly significant role in various fields.

### Veracious Challenges to Circumspect

Navigating the complex landscape of AI development requires a careful balance between transparency, innovation, and responsible implementation. While algorithmic transparency is critical for validating methods, it must be handled with caution to avoid unintended consequences and misuse. Similarly, overengineering and overregulation can stifle innovation, making it essential to strike a balance that promotes both progress and safety. Complete transparency in reporting is also vital, ensuring that all analytical processes are fully disclosed and accessible, enabling thorough evaluations of model performance and potential biases.

Algorithmic transparency is crucial for the validation of methods and tools [96]. However, it also introduces risks, such as the potential for misuse, unintended consequences, or manipulation of models and systems. Black box methods, while generally undesirable when they fail to meet expected safety and performance standards, can be advantageous in scenarios where securing the system against tampering is necessary. Moreover, as argued by some AI literature [97,98], if a well-validated black box model demonstrates a substantial statistical advantage over the best transparent model, it may be both impractical and ethically questionable to disregard its superior performance and use [98]. Navigating these trade-offs requires careful consideration and expertise.

Overengineering and overregulating pose challenges in science and technology [99]. When best practices are enforced in rigid, bureaucratic ways, there is a risk of stifling innovation and slowing progress. Additionally, disguising decision models that guide user actions as mere advisory tools is a persistent issue in health AI [12]. Regulation must address these practices, as they can render regulation ineffective and distort performance and safety requirements during the design of AI systems. Ensuring safety and performance through best practices is essential, but it must be balanced against the risks of delaying the deployment of valuable AI/ML applications in health care and health sciences. Achieving this balance is crucial for fostering both innovation and responsible implementation.

It is essential to practice complete reporting of the full process, especially all the analyses and modeling procedures applied to the data [100,101]. This would enable a thorough evaluation of the robustness and any potential issues of bias. Providing complete access to the algorithms and data, with a detailed disclosure of the entire analytical process, including all model selection and error estimation steps, would definitely help the comprehensive evaluation of model performance and reliability, and its associated aspects, such as overfitting or overconfidence.

## Discussion

### Principal Findings and Framework Synthesis

This work results in the ETEPAI, which is structured around 4 key stages: design, development, deployment, and postdeployment. It is a wholesome consideration of an AI tool production from designing, development, deployment, and postdeployment stages to be executed in a sound approach and with good adherence to the ethical principles, solid preparation, and execution that meet expected achievable usefulness, information technology feasibility, and crucial technical requirements including deployment strategy, monitoring and evaluation plan, impact assessment, financial projections for sustainability, and take into account what is cohesive to the epidemiologic research methodology of rigorous validation and complete reporting [15]. This is achieved by integrating multiple frameworks and guidelines to ensure a broad and comprehensive approach to AI tool development in health care. This corroborates the declaration of Innsbruck that underscores the evaluation of information systems in health care throughout design and implementation stages [102], and the multistep process design framework of iterative evaluation and system assessment as central to overall development planning [103], and also matches well the key challenges posed by AI in a real-world context of care and services according to the health technology assessment core model [104].

### Comparison With Existing Guidelines

Since ETEPAI is essentially a synthesized summary of existing robust recommendations, it is an evidence-based guidance on the tasks it is meant for, which may include using it as an evaluation tool in a systematic review of papers reporting on AI tools development, which would ease the task of using multiple similar checklists [105]. ETEPAI is different from other similar tools, but does not form its referenced base. Multimedia Appendix 2 tabulates the characteristics of these guidelines, checklists, and frameworks. The methodological guidelines tool reviewed the methodologies applied in 134 selected studies and developed its checklist to provide a systematic framework for estimating population-based health indicators using linked data and ML techniques [52], but lacked in certain emphasis, such as on error analysis, guidance on deployment, and postdeployment strategies. Another was based on theoretical knowledge (Clinical Artificial Intelligence Research checklist) [53], one that is discipline-specific that has yet to be developed through the proper processes (Model for Assessing the Value of Artificial Intelligence in Medical Imaging) [54], another translational-to-practice focused evaluation framework (Translational Evaluation of Health Care Artificial Intelligence framework) [55], another that is a publication appraising tool in radiology [56] to complement an existing one (Data Algorithm Training Output method) [57], another for setting specific “verification paradigms” of AI tools in clinical decision-making [58], one a function-specific (algorithm selection) [59], another that is ethics-focused [60], one forcedly matched to the sequential phases of experimental testing and clinical research for drugs and medical devices [61], while another is a reporting guideline developed through the Delphi method of 11 researchers from 3 institutions on 3 different continents, providing suggestions for every section of a journal paper from title to discussion on limitations [62]. There is another reporting guideline specific to radiomic research (Checklist for Evaluation of Radiomics Research) [63] for study planning, paper writing, and evaluation during the review process. There is one radiology AI software specification, classification, and performance evaluation framework till postdeployment, which is comprehensive but presented as fragmented parts and might complicate its application [64]. Another radiology framework (Radiology Artificial Intelligence Deployment and Assessment Rubric) emphasizes proper study designs in the assessment of its 7 preidentified function domains named as hierarchical levels of efficacy [106], and not along a clear development process of AI tools. A recent checklist, OPTICA (Organizational Perspective Checklist for Artificial Intelligence Solutions Adoption), was developed by a single institution primarily to assess completed AI solutions that are to be adopted in health care organizations [65]. It comprises 13 chapters of 3 to 12 items in each chapter to be completed by 5 main stakeholders who are assumed to be educated and able to mark off the checklist items competently and sequentially by a single identified stakeholder. This is different from ETEPAI, where full consideration is to be given by the project team at the beginning, and adhered to or referenced during the process till the end. Although OPTICA does not specifically address ethics, the 77 probing questions are indeed noteworthy to all stakeholders during adoption consideration. This is unlike ETEPAI, which mandates early and continuous adherence to ethical and procedural considerations throughout the entire AI project life cycle in general. TRIPOD-LLM (Transparent Reporting of a Multivariable Model for Individual Prognosis or Diagnosis-Large Language Models) [66] focuses on text-only large language model (LLM) projects and standardized reporting to enhance clarity, transparency, and accountability. While ETEPAI is embedded in project workflows, TRIPOD-LLM aids in appraising study quality and facilitating reproducibility with less emphasis on guiding processes from initiation to completion if used as a post hoc assessment tool. Another, which is named as Awesome AI Guidelines on GitHub, is a rich compilation of many policy documents, guidelines, and checklists related to AI, with the majority of them on ethics principles; only a few are dated beyond the year 2020 [107]. Although every evaluation framework has its respective strengths and should be used where appropriate, ETEPAI is believed to have all the pivotal aspects required of AI tools production, with an emphasis on methodology.

### Addressing Implementation and Sustainability Challenges

Recent studies highlighted that current regulatory approvals for AI models often did not ensure fairness, with bias evaluation and mitigation typically occurring postdeployment [108]. Integrating AI models into clinical practice involves significant challenges, including information technology system integration, local fine-tuning, and addressing unintended consequences such as automation bias [108]. Robust human-machine collaboration and continuous monitoring are essential to improve performance and prevent failures caused by changes in software or equipment. Postdeployment maintenance and sustainability of AI models in clinical settings present significant ongoing challenges, including model drift, data drift, concept drift, harmful feedback loops, adversarial attacks, and potential biases that may emerge in real-world applications, retraining schedules, and managing overall AI tools maintenance [109]. Additionally, usability evaluation faces obstacles such as the lack of transparency and explainability in AI systems, often referred to as “black boxes” or “black box inside a black box” nature of commercial AI models [108], which can hinder user trust and satisfaction. To address these issues, it is essential to conduct usability evaluations according to recognized standards, assess user learning curves to inform training programs, evaluate stakeholder and patient acceptability to ensure alignment with user expectations, and redesign AI systems tailored for their intended clinical use, focusing on integration into workflows, reliability, and regulatory compliance [55].

### Strategic Value and Generalizability

The ETEPAI framework is structured to proactively address these multifaceted challenges. By mandating that critical issues such as fairness, bias, and workflow integration be considered from the initial design stage, it shifts mitigation from a reactive, postdeployment afterthought to a foundational project requirement, including agentic AI uses [110]. Its dedicated postdeployment stage provides a clear roadmap for the continuous monitoring and usability evaluations essential for long-term sustainability and trust. This comprehensive, life cycle–based approach distinguishes ETEPAI from other valuable but more specialized guidelines. While built upon excellent existing frameworks such as TRIPOD-AI (Transparent Reporting of a Multivariable Prediction Model for Individual Prognosis or Diagnosis - Artificial Intelligence, a reporting guideline), FUTURE-AI (Fairness, Universality, Traceability, Usability, Robustness, and Explainability - Artificial Intelligence, imaging-focused), and ALTAI (Assessment List for Trustworthy Artificial Intelligence, an ethics checklist), ETEPAI’s novelty lies in its integration of ethical, technical, and epidemiological considerations into a single, cohesive structure. Consequently, this end-to-end structure establishes its practicality as an actionable guide for multidisciplinary teams from project inception, rather than as a post hoc reporting or evaluation checklist.

Consequently, ETEPAI is designed to be model-agnostic. Whether the underlying technology is a traditional logistic regression, a convolutional neural network, or an LLM, the necessity for ethical oversight, rigorous epidemiological study design (such as external validation), and postdeployment monitoring remains constant. While the specific performance metrics may differ between model types, such as calibration plots for regression vs red-teaming for generative artificial intelligence (GenAI), the governance checkpoints provided by ETEPAI are universally applicable steps in the translational pathway.

### Application Nuances

Notwithstanding the comprehensive and process-centric evaluation of ETEPAI, the degree of relevance and importance of ETEPAI’s items within the 4 stages of design, development, deployment, and postdeployment, and the 3 domains of ethics, technical, and epidemiological principles may differ in different AI projects of tools or systems. The different objectives, settings, and complexities of solutions of each AI tool would demand different attention on different items in ETEPAI. Nevertheless, every item requires consideration and must be justified if bypassed. When any of the items, stages, or domains raises concern, for proper understanding and evaluation, we are to refer to the original guideline or checklist (as given in the parentheses in the footnote to Table 1).

There are a few potential drawbacks of ETEPAI arising from the recommendations it is built on and the evolving techniques and technology of AI. Some of these include a lack of detail on the class imbalance mitigation strategy, approaches to address fairness, heterogeneity in estimates of model parameter values, and model performance. Additionally, further guidance will be needed on specific AI areas such as AI tools to influence social and health behaviors at scale in social media and different platforms, artificial general intelligence in health care, and other guides that were developed for specific purposes, as alluded to in this discussion, such as the OPTICA tool in assessing AI solutions in health care settings [65]. This is because ETEPAI is mainly for sign-posting the important considerations as necessary indicators and not for instructing the proper means of achieving them, which are dependent on the dataset’s quality and distribution. However, ETEPAI reminds developers and stakeholders to provide a clear explanation and reporting of all decisions taken during the whole process, from development to postdeployment. Guidance on advanced evaluation methods, such as handling Pareto frontiers for multiobjective optimization (balancing fairness and accuracy) [111,112], would require more elaborative and extensive explanation beyond the foundational structure ETEPAI aims to provide.

### Limitations

While comprehensive, the ETEPAI may not fully address all aspects of certain types of AI models, especially newer or more complex models such as GenAI or agentic AI where multiple AI tools are developed and deployed to complement collaboratively [113], or in other sectors than health care such as education [114,115] and research enterprise [116,117], which are evolving and with robust evaluation frameworks that are yet available [118]. The POLARIS-GM (Partnership for Oversight, Leadership, and Accountability in Regulating Intelligent Systems–Generative Models in Medicine) [118] is an ongoing initiative focused on developing granular, scenario-based regulatory guidance for the unique challenges of GenAI and LLMs in medicine, using a multiphase, consensus-driven approach to generate new recommendations for postimplementation controls. In contrast, ETEPAI serves as a broad, comprehensive guide and evaluation tool for general AI tool production, synthesizing existing recommendations into practical pointers. We acknowledge that certain technical specifics of traditional ML do not map directly to GenAI, such as the standard calibration metrics, discrimination measures such as receiver operating characteristic area under curve, or the evaluation of feature stability, which are often replaced in GenAI by semantic similarity scores and human-preference alignment, and shift toward detecting toxicity and protecting against jailbreaking attempts, respectively. However, ETEPAI’s core principles remain applicable if adapted appropriately. These and the stages of considerations are still relevant to GenAI and agentic AI across their life cycle [119], from defining a safe operational domain in the design stage, and conducting adversarial “red-teaming” and error analysis to evaluate hallucination rates and consistency in the development stage, to mitigating automation bias through stakeholder education during the deployment stage and continuously monitoring for emergent harms in the postdeployment stage. While ETEPAI provides the overarching governance structure, developers must supplement it with specific, evolving guidelines for generative models, which incorporate initiatives such as the TRIPOD-LLM and the POLARIS-GM, to address their unique technical and ethical challenges.

When the referenced materials are used, the interpretation of criteria for items relies on expert ratings, which can introduce subjectivity and potential bias, leading to inconsistencies in the evaluation of AI tools across different studies and users. As this study is primarily on assimilating existing recommended materials to produce one overarching guidance and not on evaluating the quality of the referenced materials, there is neither analysis of their processes and products, nor were comparisons made among them. However, ETEPAI uses a consensus-driven approach by using the collective judgment of multiple experts, which balances individual biases and ensures a more reliable and balanced assessment. Additionally, it emphasizes transparency by documenting the criteria and rationale behind the ratings. This openness allows for thorough scrutiny and review, helping to identify and address potential biases or inconsistencies in the evaluation process.

All care was taken to screen through eligible guides and checklists at the time of publication of ETEPAI. There are many similarities among the included and excluded guides, but only those that are considered to be robust in development and focus on the process from design to postdeployment formed the basis of ETEPAI. However, the content of ETEPAI is noted to be inclusive of almost all important considerations in the excluded guides, as discussed. The similarity of ETEPAI to many guidelines and checklists is intended as per the method used to create it. As ETEPAI draws its validity from 30 robust, consensus-derived guidelines it synthesizes, this provides a strong evidence base for ETEPAI’s applicability across diverse AI models. Although the acceptability, applicability, and effectiveness of ETEPAI in real-world uses have not been tested, it is expected to be similar to those that were robustly tested. Although ETEPAI’s content validity is established through its systematic synthesis of existing, evidence-based, and widely accepted guidelines, making it conceptually sound upon proposal, it would require subsequent empirical work to confirm them. This may include methods such as (1) expert consensus: conducting a formal Delphi study with international experts in clinical AI, ethics, and epidemiology to refine and weigh the framework’s items; (2) pilot testing: implementing the ETEPAI framework within one or more real-world AI development projects to gather qualitative feedback on its practicality and utility; and (3) retrospective application: using ETEPAI as an evaluation tool for a cohort of published AI studies to assess its applicability and identify areas for improvement. While ETEPAI as a unified entity awaits prospective empirical testing, its component “data points,” the individual requirements for fairness, robustness, and reporting, are already established standards in the scientific community.

Despite its comprehensive nature, the ETEPAI may still be challenging for some stakeholders to fully grasp without sufficient training or motivation. This is evidenced by the tool’s expansive supplementary materials for full explanations, which may limit its immediate usability, as users need to refer to additional resources for a lucid understanding. Many pointers in the footnotes are cross-disciplinary and could be beyond any project team member. For example, the emphasis on diversity in the datasets and caution on using existing datasets that might limit the representativeness or comprehensiveness necessary for some AI tool development scenarios are to be appreciated by all stakeholders and upheld by methodologists or epidemiologists to ensure compliance throughout the whole production process. Although the framework highlights the need for rigorous external validation and adherence to reporting guidelines, these processes can be resource-intensive and may not be feasible for all projects. Furthermore, the rapidly evolving nature of AI technology, AI models updating methods [120], and health care practices may necessitate frequent updates to the ETEPAI framework to maintain its relevance and effectiveness. Public scrutiny and transparency are important. Publishing project protocols or making them available on public platforms can help ensure that the best practices recommended by ETEPAI are followed. It also makes it easier to explain and document any changes or deviations from the proposed design.

### Conclusions

The ETEPAI is not just another guideline, but it combines existing robustly developed policies, guidelines, and checklists in one place that map out the ideal route, navigational aids, and risky areas throughout the whole AI tools development process. By focusing on its core function as a guide, this synthesized guideline encourages thoughtful application, ensuring its adaptability to diverse settings while avoiding misuse as a rigid standard, evaluation benchmark, or risk assessment framework. The guideline provides flexible principles and recommendations that must be adapted to specific contexts rather than being followed rigidly or universally applied. While it promotes quality, it does not include structured criteria or metrics for evaluating performance or outcomes. The guideline does not focus on identifying, quantifying, or mitigating risks but may complement such tools when integrated appropriately. Therefore, ETEPAI should be more useful as a training reference manual than a quality standard reference manual. This is because to appreciate and to put it to immediate practice requires previous training on AI concepts and technologies. It is possible to use ETEPAI as a template for training purposes to impart AI-related clinical competencies in health care professionals [121]. It is both comprehensive and complete, containing almost all critical considerations, as well as briefly and crisply presenting those considerations in a table of probing questions and another table of critical pointers in 3 domains of ethics, technical, and epidemiological principles when considering an AI tool.

Applying ETEPAI ensures comprehensive guidance and encourages a multidisciplinary approach to AI tool development, from design through postdeployment. This framework promotes adherence to ethical standards, robust technical execution, and rigorous epidemiological research methodologies. By integrating these considerations, AI tools can achieve their intended impact in health care settings, offering reliable, effective, and ethically sound solutions. To ensure effective application of ETEPAI and meticulous attention to the aspects throughout the AI development life cycle, it is essential for users to understand each and every included guideline, checklist, assessment, and framework, and be familiar with the ethical and regulatory concepts. We highly advocate its use to ensure that developers and authors appropriately design and comprehensively report AI work leading to high-quality, practical AI applications that align with clinical needs and ethical imperatives. ETEPAI’s product-centric approach may effectively align with EU trustworthiness benchmarks on safety, robustness, and ethics from design to deployment [13]. This helps the final AI products to be compliant with the EU’s requirements for the protections of health, safety, fundamental rights, and comprehensive risk management that considers impacts on both products and systems [75]. Using ETEPAI should be easy and sufficient for experienced professionals and serve as clear signposts for less prepared stakeholders. As the field of AI continues to evolve with new technologies and algorithms, we anticipate ongoing updates to ETEPAI as new guidelines and reporting standards emerge to ensure high-quality scientific research and the ethical application of AI in medicine.

## Supplementary material

10.2196/80340 Multimedia Appendix 1 Table S1: recommended guidelines, checklists, assessment frameworks, and recommendations, and Table S2: AI ethics, safety, and dataset diversity policy frameworks (descending orders of relevancy and recentness). AI: artificial intelligence.

10.2196/80340 Multimedia Appendix 2 Table S3: characteristics of guidelines, checklists, and frameworks related to AI tools (according to the alphabetical and ascending year of the publication). AI: artificial intelligence.

10.2196/80340 Multimedia Appendix 3 Outline of an AI research proposal. AI: artificial intelligence.

10.2196/80340 Multimedia Appendix 4 Further footnotes to Table 2.

10.2196/80340 Checklist 1 PRISMA-ScR checklist.

## Abbreviations

AI: artificial intelligence
AIPA: Artificial Intelligence Prediction Algorithm
ALTAI: Assessment List for Trustworthy Artificial Intelligence
ETEPAI: ethical, technical, and epidemiological considerations for all involved in artificial intelligence tool production
EU: European Union
FURM: Fair, Useful, the Reliable Artificial Inelligence Model
FUTURE-AI: Fairness, Universality, Traceability, Usability, Robustness, and Explainability - Artificial Intelligence
GenAI: generative artificial intelligence
HIPAA: Health Insurance Portability and Accountability Act
LLM: large language model
ML: machine learning
OPTICA: Organizational Perspective Checklist for Artificial Intelligence Solutions Adoption
PE/ICOTS: Population or Participants, Exposure/Intervention, Comparator, Outcomes, Timing, and Setting
POLARIS-GM: Partnership for Oversight, Leadership, and Accountability in Regulating Intelligent Systems–Generative Models in Medicine
PRISMA-ScR: Preferred Reporting Items for Systematic Reviews and Meta-Analyses extension for Scoping Reviews
STANDING: Standards for Data Diversity, Inclusivity, and Generalisability
SUDO: pseudo-label discrepancy
TRIPOD-AI: Transparent Reporting of a Multivariable Prediction Model for Individual Prognosis or Diagnosis - Artificial Intelligence
TRIPOD-LLM: Transparent Reporting of a Multivariable Model for Individual Prognosis or Diagnosis - Large Language Models
