# Supplementary material for: Important Ethical, Technical, and Epidemiological Considerations in an AI Tool Production (ETEPAI): Scoping Review
Source: JMIR AI. 2026 Mar 5;5:e80340. doi: 10.2196/80340 (PMC12977167; doi:10.2196/80340)
Supplement: Multimedia Appendix 1 — Table S1: recommended guidelines, checklists, assessment frameworks, and recommendations, and Table S2: AI ethics, safety, and dataset diversity policy frameworks (descending orders of relevancy and recentness). AI: artificial intelligence. [file ai-v5-e80340-s001.pdf]

Table S1: recommended guidelines, checklists and assessment frameworks and recommendations

No.	Recommendation / Type* / Checklist	Content
1.	AIPA [1]/ Technical/ In form of narrative without a checklist	AIPA (Artificial Intelligence Prediction Algorithm) provides a comprehensive guideline for the development, validation, evaluation, and implementation of AI tools in healthcare. It outlines six key phases starting with data collection and management, progressing through model development, validation, software development, impact assessment, and culminating in implementation and monitoring in clinical practice.
2.	APPRAISE-AI [2] / Technical / eTable 1: The APPRAISE-AI Tool to Assess Quality of AI Studies in Medicine;	This evaluates quality of AI studies in the model development process across 6 domains: 1) clinical relevance, 2) data quality, 3) methodological conduct, 4) robustness of results, 5) reporting quality, and 6) reproducibility in terms of transparency and data sharing. These domains include 24 items with a maximum overall score of 100 points. Higher points indicating stronger methodological or reporting quality.
3.	CODE-HER [3] / Reporting / Best practice checklist	The CODE-EHR Minimum Standards Framework aims to improve the design and reporting of research studies using structured electronic health-care data. It requests for clarity on reporting and defines a set of minimum and preferred standards for the processes involved in 1) coding, dataset construction and linkage, 2) details and transparency of the preceding step, 3) disease and outcome definitions, 4) analysis, and 5) research governance which emphasises on patient and public engagement throughout the development process. Researchers are advised to use this checklist in the design phase of their study to ensure that important criteria for successful research and research impact are being used.
4.	DECIDE-AI [4] / Reporting / Item Checklist	The DECIDE-AI (Developmental and Exploratory Clinical Investigations of DEcision support systems driven by Artificial Intelligence) comprises key items to be reported in early-stage clinical studies of AI-based decision support systems in healthcare to facilitate the appraisal of these studies and replicability of their findings. It has 17 AI-specific reporting items (with 28 subitems) and 10 generic reporting items with a paragraph for explanation for each of this item. DECIDE-AI focuses on the interaction between humans and AI systems, emphasizing "augmented intelligence." Intended for early-stage clinical studies, these guidelines complement existing ones by addressing human-AI interaction before clinical validation. This approach aims to facilitate the efficient translation of AI algorithms from development to clinical use.
5.	SPIRIT-AI [5] / Reporting / Checklist (web interactive and Word)	The SPIRIT-AI (Standard Protocol Items: Recommendations for Interventional Trials–Artificial Intelligence) extension is a reporting guideline for clinical trial protocols evaluating interventions with an AI component. It includes 15 (12 extensions, 3 elaborations) new items in addition to the core SPIRIT 2013 of 33 items. SPIRIT-AI requires clear descriptions of the AI intervention including instructions and skills needed for use, the setting in which the AI intervention will be integrated, considerations for the handling of input and output data, the human-AI interaction and analysis of error cases.

6.	CONSORT-AI [6] / Reporting / Checklist (web interactive and Word)	The CONSORT-AI (Consolidated Standards of Reporting Trials for reporting AI studies) includes 14 (11 extensions, 3 elaborations) new items in addition to the core CONSORT 2010 items. It recommends investigators to provide clear descriptions of the AI intervention, including instructions and skills required for use, the setting in which the AI intervention is integrated, the handling of inputs and outputs of the AI intervention, the human-AI interaction and providing analysis of error cases.
7.	The TRIPOD [7] and TRIPOD-AI [8] / Reporting / **Supplementary Table 1:** The Expanded Checklist (PDF) (Explanation & Elaboration)	The TRIPOD (Transparent reporting of a multivariable prediction model for individual prognosis or diagnosis) is a checklist of 22 items, deemed essential for transparent reporting of a prediction model study. TRIPOD-AI assists in reporting research in which a multivariable prediction model is being developed (or updated) or validated (tested) using any (supervised) ML technique. The checklists are not a quality appraisal tool.
8.	PROBAST [9] and PROBAST-AI [10,11] / Reporting / **Supplementary Table 3**: The steps for use of the PROBAST+AI tool; **Supplementary Table 4:** PROBAST+AI Explanation	The PROBAST (Prediction model Risk Of Bias ASsessment Tool) comprises four domains (participants, predictors, outcome and analysis) and contains 20 signalling questions to facilitate risk of bias assessment of prediction model studies from the study design, conduct to analysis. PROBAST-AI comprises two components: model development and model evaluation. In model development, users assess quality and applicability with 16 targeted signalling questions. While model evaluation uses 18 targeted questions to assess risk of bias and applicability. Both components share four domains: participants and data sources, predictors, outcome, and analysis, with the prediction model's applicability specifically rated in the first three domains.
9.	STARD-AI [12,13] / Reporting / **Supplementary Table 2** Downloadable version of the STARD-AI checklist	The STARD-AI (Standards for Reporting of Diagnostic Accuracy Studies AI Extension) is used to report diagnostic accuracy/test studies. STARD-AI is a reporting guideline created for diagnostic accuracy studies involving artificial intelligence. As an extension of the STARD 2015 checklist, it introduces 18 new or modified items to address unique AI-specific considerations (total 40 items). The guideline encourages authors to detail dataset practices, AI model evaluation, algorithmic bias, and fairness, promoting transparent reporting, which helps key stakeholders evaluate the bias, applicability, and generalizability of study findings.
10.	MINIMAR [14] / Reporting / **Table 1:** Reporting standards for 4 essential components of artificial intelligence solutions in health care	The MINIMAR (MINimum Information for Medical AI Reporting) guides on the minimum information necessary to understand intended predictions, target populations, and hidden biases, and the ability to generalize these emerging technologies in four sections: (1) information on the population providing the training data, (2) training data demographics (3) detailed information about the model architecture and development, and (4) model evaluation, optimization, and validation to clarify how local model optimization can be achieved and enable replication and resource sharing. These guidelines suggest how to report information in four key areas, with overlap between MINNIMAR and MI-CLAIM, both focusing on AI algorithm development and validation.

11.	CLAIM [15] / Reporting / Checklist	The CLAIM (Checklist for Artificial Intelligence in Medical Imaging) is modelled after the STARD guideline and has been extended to address applications of AI in medical imaging that include classification, image reconstruction, text analysis, and workflow optimization to guide complete reporting of research.
12.	MI-CLAIM [16] / Reporting / Checklist	The MI-CLAIM (Minimum information about clinical artificial intelligence modelling) is a tool to improve transparent reporting of AI algorithms in medicine. It aims to enable a direct assessment of clinical impact including fairness and bias, and secondly to allow rapid replication of the technical design process of any legitimate clinical AI study. The six parts are: 1) study design comprises clinical setting, performance measures, population composition, and current baselines to measure performance against, 2) data partitions for model training and testing, 3) optimisation and final model selection, 4) performance evaluation to be reported at the model itself, and the model's clinical performance metrics, 5) model examination as a 'sanity check', to uncover biases, to understand model behaviour, 6) reproducible pipeline by complete sharing of the code. This guideline focuses more on reporting details about the AI algorithm itself rather than its application in specific studies. They complement AI-specific extensions like SPIRIT-AI, CONSORT-AI, STARD-AI, and TRIPOD-AI, providing additional value when used together.
13.	CHEERS-AI [17,18] / Reporting / **Table 1:** Items included in CHEERS-AI reporting guideline extension	The CHEERS-AI (Consolidated Health Economic Evaluation Reporting Standards-AI) assists in describing health economic evaluations to estimate the value for money (cost effectiveness) of AI interventions.
14.	IDEAL [19,20] / Reporting / **Table 2:** IDEAL Checklists	The IDEAL (Innovation, Development, Exploration, Assessment, and Long-term) Framework describes the five stages through which surgical therapy innovation normally passes. Each IDEAL stage is defined by key research questions which are intended to provide a minimum list of concepts authors should include in a report of surgical and device innovation. It can also be used both prospectively to help plan a study and retrospectively to assist in appraisal. IDEAL-D Framework for Device Innovation is a consensus statement on the preclinical stage of development (Stage 0)[21].
15.	FUTURE-AI [22] / Technical / Table 1: Overview of the FUTURE-AI checklist	The guiding principles of FUTURE-AI (Fairness, Universality, Traceability, Usability, Robustness and Explainability) are 1) Fairness, 2) Universality, 3) Traceability, 4) Usability, 5) Robustness and 6) Explainability appeared as the quality check questionnaire comprises 55 practical questions which as a whole summarise the 5 guiding principles and encapsulate each of their recommendations. They aim to guide developers, evaluators and other stakeholders in delivering medical AI tools in health imaging that are trustworthy and optimised for real-world practice.
16.	ALTAI [23,24] / Technical / Trustworthy AI Assessment List (web-based and document-based)	The ALTAI (Assessment List for Trustworthy AI) provided by the European Commission's High-Level Expert group for Artificial Intelligence. It comprises seven requirements for Trustworthy AI: 1) human agency and oversight, 2) technical robustness and safety, 3) privacy and data governance, 4) transparency, 5) diversity, non-discrimination and fairness, 6) societal and environmental well-being and 7) accountability, and 60 questions in total.

17.	The medical algorithmic audit [25] / Technical / **Figure 1:** Overview of the medical algorithmic audit and **Table 1:** Actions for developers and users at each stage of the medical algorithmic audit	The audit framework for medical algorithms consists of several key phases: scoping, mapping, artifact collection, testing, and reflection. In the scoping phase, the audit's scope and intended impacts are defined, considering ethical and clinical motivations. The mapping phase involves identifying stakeholders, resources, and risks, along with understanding the algorithm's integration into clinical workflows. Artifact collection gathers all relevant documentation, datasets, model information, and previous evaluations. The testing phase includes exploratory error analysis, subgroup testing, and adversarial testing to uncover algorithmic errors and performance issues. Finally, the reflection phase assesses the results, proposes risk mitigation strategies, and makes recommendations for continued or modified use of the algorithm.
18.	SUDO framework [26] / Technical / Overview of the SUDO framework	The SUDO (pseudo-label discrepancy overview) is a framework for identifying unreliable AI predictions, selecting favourable AI systems, and assessing algorithmic bias without ground-truth labels. It involves deploying a probabilistic AI system, generating output distributions, pseudo-labelling samples, training classifiers, and evaluating performance discrepancies to detect class contamination and validate pseudo-labels.
19.	FURM assessment [27] / Technical / **Appendix**: FURM Assessment Report Templates & Examples	The FURM (Fair, Useful, and Reliable AI Model) assessment was developed by the Data Science team at Stanford Health Care as a testing and evaluation mechanism to identify fair, useful and reliable AI models by conducting an ethical review to identify potential value mismatches, running simulations to estimate usefulness, financial projections to assess sustainability, analyses to determine IT feasibility, designing a deployment strategy, and recommending a prospective monitoring and evaluation plan. Each FURM assessment consists of 3 stages to evaluate the "what & why" (Stage 1) motivating a particular AI use case, "how" (Stage 2) a given AI model will be formulated, evaluated and integrated into a given healthcare system workflow, and the impact of the proposed implementation (Stage 3). Each stage addresses a different aspect of the use case.

*Technical or reporting guideline

Some reporting guidelines are study design specific (TRIPOD-AI for prognostic and diagnostic studies, STARD-AI for diagnostic test studies, SPIRIT/CONSORT and SPIRIT/CONSORT-AI are for clinical trials), stage specific (DECIDE-AI for early clinical studies) or discipline specific (CHEERS-AI for health economy, IDEAL for surgery, and CLAIM and FUTURE-AI for radiology)[4].

Table S2: AI ethics, safety and dataset diversity policy frameworks (descending orders of relevancy and recentness)

No.	Name/Organisation/Year	Core Value/Principle	Content/Policy
1.	**Recommendation on the Ethics of Artificial Intelligence** / UNESCO / 2022 [28]	This is a standard-setting instrument developed through a global approach for all authorities, bodies, research and academic organisations, and institutions in public, private and civil society sectors involved in AI technologies, so that the development and use of AI technologies are guided by both sound scientific research as well as ethical analysis and evaluation. This document is based on international law proposing 4 core values and 10 core principles laying out a human-rights centred approach to the ethics of AI:<br>Four core values as the foundations for AI systems that work for the good of humanity, individuals, societies and the environment:<br>1. Human rights and human dignity<br>2. Living in peace<br>3. Ensuring diversity and inclusiveness<br>4. Environment and ecosystem that flourish<br>Ten core principles:<br>1. Proportionality and do no harm<br>2. Safety and security<br>3. Right to privacy and data protection<br>4. Multi-stakeholder and adaptive governance & collaboration<br>5. Responsibility and accountability<br>6. Transparency and explainability<br>7. Human oversight and determination<br>8. Sustainability impact<br>9. Awareness & literacy of the public<br>10. Fairness and non-discrimination	Eleven areas of policy action:<br>1: Ethical impact assessment (see below)<br>2: Ethical governance and stewardship<br>3: Data policy<br>4: Development and international cooperation<br>5: Environment and ecosystems protection<br>6: Gender<br>7: Culture<br>8: Education and research<br>9: Communication and information<br>10: Economy and labour<br>11: Health and social well-being<br><br>Readiness Assessment Methodology (RAM) questionnaire includes a range of quantitative and qualitative questions designed to gather information about different dimensions related to a country's AI ecosystem including the legal and regulatory, social and cultural, economic, scientific and educational, and technological and infrastructural dimensions [29].<br><br>The Ethical Impact Assessment (EIA) considers the entire process of designing, developing and deploying an AI system allowing for assessment of the risks before and after the system is released to the public [30].
2.	**UN Resolution on AI** / 2024 [31]	Adopting a US-led draft resolution without a vote, this UN document highlights the respect, protection and promotion of human rights in the design, development,	UN resolves to:<br>1. bridge the AI and other digital divides between and within countries.

No.	Name/Organisation/Year	Core Value/Principle	Content/Policy
		deployment and the use of AI. The text was "co-sponsored" or backed by more than 120 Member States. The General Assembly also recognised AI systems' potential to accelerate and enable progress towards reaching the 17 SDGs.<br><br>The purpose of this resolution is to seize the opportunities of safe, secure and trustworthy non-military AI systems for sustainable development. The AI life cycle includes the stages: pre-design, design, development, evaluation, testing, deployment, use, sale, procurement, operation and decommissioning. The principles are human-centric, reliable, explainable, ethical, inclusive, in full respect, promotion and protection of human rights and international law, privacy preserving, sustainable development oriented, and responsible in the three important dimensions of economy, social and environment in a balanced and integrated manner that promote digital transformation, peace and overcome digital divides between and within countries; and promote and protect the enjoyment of human rights and fundamental freedoms for all, while keeping the human person at the centre.	2. promote safe, secure and trustworthy AI systems to accelerate progress towards the full realization of the 2030 Agenda for Sustainable Development.<br>3. encourage Member States and invites multi-stakeholders from all regions and countries, including from the private sector, international and regional organizations, civil society, the media, academia and research institutions and technical communities and individuals, to develop and support regulatory and governance approaches and frameworks related to safe, secure and trustworthy AI systems.<br>4. call upon Member States and invites other stakeholders to take action to cooperate with and provide assistance to developing countries towards inclusive and equitable access to the benefits of digital transformation and safe, secure and trustworthy AI systems.<br>5. emphasise that human rights and fundamental freedoms must be respected, protected and promoted throughout the life cycle of AI systems.<br>6. encourage all Member States, where appropriate, in line with their national priorities and circumstances to promote safe, secure and trustworthy AI systems in an inclusive and equitable manner, and foster an enabling environment for such systems to address the world's greatest challenges, including achieving sustainable development in its three dimensions – economic, social and environmental.<br>7. recognise that data is fundamental to the development and operation of AI systems that the fair, inclusive, responsible and effective data governance, improving data generation, accessibility and infrastructure, and the use of digital public goods are essential to harnessing the potential of safe, secure and trustworthy AI systems.<br>8. acknowledge the importance of continuing the discussion on developments of AI governance so that international approaches keep pace with the evolution of AI systems and

No.	Name/Organisation/Year	Core Value/Principle	Content/Policy
			their uses; and encourage continued efforts by the international community to promote inclusive research, mapping and analysis that benefit all parties on the potential impacts and applications that AI systems and rapid technological bring; and to inform how to develop, promote and implement effective, internationally interoperable safeguards, practices, standards and tools for AI designers, developers, evaluators, deployers, users and other stakeholders for safe, secure and trustworthy AI systems; and stresses the need for all stakeholders to continue to work together for more cohesive, effective, coordinated and inclusive engagement and participation of all communities, particularly from developing countries, in the inclusive governance of safe, secure and trustworthy AI systems.<br>9. encourage the private sector to adhere to applicable international and domestic laws and act; recognise the need for increased collaboration including between and within the public and private sectors and civil society, academia and research institutions and technical communities to provide and promote fair, open, inclusive and non-discriminatory business environment, economic and commercial activities, competitive ecosystems and marketplaces across the life cycle of safe, secure and trustworthy AI; encourage Member States to develop policies and regulations to promote competition in safe, secure and trustworthy AI systems and related technologies by supporting and enabling new opportunities for small businesses and entrepreneurs and technical talent, and enabling fair competition in the AI marketplace, through critical investment, especially for developing countries.<br>10. call upon specialised agencies, funds, programmes, other entities, bodies and offices, and related organizations of the UN system, within their respective mandates and resources, to continue to assess and enhance their response to leverage

No.	Name/Organisation/Year	Core Value/Principle	Content/Policy
			the opportunities and address the challenges posed by AI systems in a collaborative, coordinated and inclusive manner, through appropriate inter-agency mechanisms, including by conducting research, mapping and analysis that benefit all parties on the potential impacts and applications.
3.	**International Scientific Report on the Safety of Advanced AI: Interim Report** / 2024 [32]	This interim report brought together 75 artificial intelligence (AI) experts, including an international Expert Advisory Panel nominated by 30 countries, the European Union (EU), and the United Nations (UN), and other world-leading experts, to provide a shared scientific, evidence-based foundation about general-purpose AI (GPAI) safety. It focuses on advanced AI ie. GPAI that can perform a wide variety of tasks. It strives to understand GPAI's capabilities and risks presently and in the future, to delineate evaluation and technical methods for assessing and mitigating risks identify these risks and. It does not aim to comprehensively assess all its possible societal impacts and potential benefits. The future of GPAI technology is uncertain, with a wide range of trajectories appearing possible even in the near future, including both very positive and very negative outcomes. But nothing about the future of AI is inevitable. It will be the decisions of societies and governments that will determine the future of AI. This interim report aims to facilitate constructive discussion about these decisions.<br><br>The key question continues to be discussed is "Will continued 'scaling' of resources and refining existing techniques be sufficient to yield rapid progress and solve issues such as reliability and factual accuracy, or are new research breakthroughs required to substantially advance GPAI abilities?	This report classifies general-purpose AI risks into three categories: malicious use risks, risks from malfunctions, systemic risks, and several cross-cutting factors that contribute to many risks.<br>**Malicious use**: Fake content generated in scale and sophistication of scams and fraud, for example through 'phishing' attacks. Also, fake compromising content featuring individuals without their consent, such as non-consensual deepfake pornography. Disinformation and manipulation of public opinion, social engineering cyber-attacks, and development and malicious use of weapons, such as biological weapons are other risks.<br>**Risks from malfunctions:** Even when users have no intention to cause harm, serious risks can arise due to the malfunctioning of GPAI. These include miscommunication or misleading advertising, biased with respect to race, gender, culture, age, and disability especially harmful in high-stakes domains such as healthcare, job recruitment, and financial lending, and minute possibility of 'loss of control' scenarios with autonomous AI-systems that can act, plan, and pursue goals.<br>**Systemic risks:** These range from potential labour market impacts, AI technology divide, to privacy risks resulting from adversarial inputs where training data containing information about individuals are extracted, and environmental effects from increased CO_2 emissions and water consumption, potential copyright infringements and unclear copyright regime disincentivises GPAI developers from declaring what data they use and makes it unclear what protections are afforded to

No.	Name/Organisation/Year	Core Value/Principle	Content/Policy
			creators whose work is used without their consent to train the GPAI models. This complicates explainability and interpretability.<br>**Cross-cutting risk factors:** The characteristics of GPAI increase the probability or severity of not one but several risks:<br>• ***Technical cross-cutting risk factors*** include the difficulty of ensuring that GPAI systems reliably behave as intended, our lack of understanding of their inner workings, and the ongoing development of general-purpose AI 'agents' which can act autonomously with reduced oversight.<br>• ***Societal cross-cutting risk factors*** include the potential disparity between the pace of technological progress and the pace of a regulatory response, as well as competitive incentives for AI developers to release products quickly, potentially at the cost of thorough risk management.<br><br>Technical approaches can help mitigate risks:<br>1. Developers can train models to be more robust to inputs that are designed to make them fail ('adversarial training').<br>2. Monitor system actions, and evaluating performance once a GPAI system has been deployed.<br>3. Trading off fairness with accuracy and privacy, use of sensitive personal data in training, and reaching a balance between knowledge, meaningful and undesirable bias in the outputs.

No.	Name/Organisation/Year	Core Value/Principle	Content/Policy
4.	**STANdards for data Diversity, INclusivity and Generalisability)** / STANDING Together project team / 2023 [33]	STANDING Together aims to ensure that inclusivity and diversity are considered when developing health datasets and AI health technologies. The recommendations were established through an international consensus process, which provide guidance on transparency around 'who' is represented in the data, 'how' people are represented, and how data is used when developing AI technologies for healthcare. By getting the data foundation right, it ensures that 'no-one is left behind' as the benefits of AI in healthcare is balanced against the risks posed by algorithmic bias and harms minimising health inequalities.	**Part 1: Recommendations for Documentation of Health Datasets**<br>1. Dataset summary<br>2. Dataset identity and access<br>3. Reasons behind dataset creation and its purpose(s)<br>4. Data Origin<br>5. Data sampling and aggregation from multiple sources<br>6. Data shifts over time<br>7. Composition of groups within the dataset<br>8. Recording of Individuals' Attributes<br>9. Groups at risk of disparate health outcomes<br>10. Limitations of the dataset<br>11. Modifications made to the data<br>12. Missing data<br>13. Known or potential bias caused or exacerbated by data acquisition and processing<br>14. Known or potential exclusion introduced by data collection<br>15. Known or potential bias in assigned or derived labels<br>16. Ethics and governance<br>17. Patient and public participation<br>18. Bias and impact assessments<br>**Part 2: Recommendations for Use of Health Datasets**<br>1. Provide sufficient information about dataset(s) to allow traceability and auditability<br>2. Identify *Contextualised Groups of Interest* in advance who may be at risk of disparate performance or harm from the AI health technology<br>3. Justify that datasets have been used appropriately to support the *Intended Use Population* and *Intended Use* of the AI health technology<br>4. Report the explicit and implicit use of relevant attributes during the lifecycle of the AI health technology

No.	Name/Organisation/Year	Core Value/Principle	Content/Policy
			5. Evaluate performance of the AI health technology for contextualised groups of interest<br>6. Identify disparate performance in any additional groups outside of the pre-specified contextualised groups of interest<br>7. Report any approaches or methods (including fairness methods) used to intentionally modify performance across groups<br>8. Report limitations of datasets used, and any implications on the AI health technology<br>9. Report differences between the intended purposes of the AI health technology and datasets used, including the implications of discordance<br>10. Report findings from pre-existing assessments of the AI health technology and any datasets used<br>11. Address uncertainties and risks with mitigation plans<br><br>*Contextualised groups of interest*: groups identified in advance who may be at risk of disparate health outcomes when the AI health technology is used. These groups are defined by shared Relevant Attributes which have known or suspected associations with disparate health outcomes related to the intended use of an AI health technology.<br><br>*Intended Use Population*: The population for whom an AI health technology may be used, as pre-specified by the manufacturer.<br><br>*Intended use* (also known as Intended Purpose): The purpose for which an AI health technology may be used, as prespecified by the manufacturer or person/organisation legally responsible.

No.	Name/Organisation/Year	Core Value/Principle	Content/Policy
5.	Voluntary Code of Conduct on the Responsible Development and Management of Advanced Generative AI Systems & Artificial Intelligence and Data Act (AIDA) / September 2023 [34]	Under the AIDA, businesses will be held responsible for the AI activities under their control. They will be required to implement new governance mechanisms and policies that will consider and address the risks of their AI system and give users enough information to make informed decisions.<br><br>The AIDA will introduce new requirements for businesses to ensure the safety and fairness of high-impact AI systems every step of the way:<br>• **Design:** Businesses will be required to identify and address the risks of their AI system with regard to harm and bias and to keep relevant records.<br>• **Development:** Businesses will be required to assess the intended uses and limitations of their AI system and make sure users understand them.<br>• **Deployment:** Businesses will be required to put in place appropriate risk mitigation strategies and ensure systems are continually monitored.<br><br>The idea is to have a flexible policy, where safety obligations are tailored to the type of AI systems. The more risks are associated with an AI system, the more obligations there will be.	Key Content and Headings of the Voluntary Code of Conduct for Generative AI:<br>1. **Introduction**<br>2. **Purpose of the Code**<br>• To mitigate risks in the absence of binding regulation.<br>• Applicable to developers and managers of generative AI systems.<br>3. **Core Outcomes**<br>• **Accountability**: Establish risk management systems.<br>• **Safety**: Risk assessments and mitigation before deployment.<br>• **Fairness and Equity**: Address biases and fairness issues.<br>• **Transparency**: Inform users and experts about system capabilities and limitations.<br>• **Human Oversight and Monitoring**: Post-deployment monitoring for risks.<br>• **Validity and Robustness**: Ensure system reliability and security.<br>4. **Commitment to Responsible AI Development**<br>• Collaboration with stakeholders.<br>• Promote standards and education.<br>• Support sustainable and inclusive growth.<br>5. **Measures for Advanced Generative AI**<br>• **Accountability**: Risk frameworks, third-party audits.<br>• **Safety**: Safeguards against malicious use.<br>• **Fairness**: Dataset curation, bias testing.<br>• **Transparency**: Information on training data, watermarking.<br>• **Human Oversight**: Incident databases, usage controls.<br>• **Validity and Robustness**: Adversarial testing, cybersecurity measures.<br>6. **Signatories**<br>• Lists companies and organizations committed to the Code.

No.	Name/Organisation/Year	Core Value/Principle	Content/Policy
6.	UK's Department of Health & Social Care Guidance: **A guide to good practice for digital and data-driven health technologies** / Updated 19 January 2021 [35]	This guide is designed to support innovators in understanding what the NHS is looking for when it buys digital and data-driven technology for use in health and care, so that these principles of good practice can be built into the strategy and product development 'by design'. This, in turn, will mean that when products are presented for assessment or procurement, many of the criteria in the specification will have already been met. The intention is to smooth the path between development and procurement so that the NHS may realise the benefits that digital technologies can bring.<br><br>The National Institute for Health and Care Excellence (NICE) in collaboration with stakeholders including NHS England, NHS Digital, MedCity, Public Health England, and DigitalHealth.London, has developed the **META tool** and an **Evidence Standards Framework** to guide digital health technology developers on generating appropriate evidence for commissioners and evaluators. Digital tools are categorized into **tiers** based on their clinical impact and risk, with evidence requirements increasing as risk grows. For technologies with multiple functions, the highest-risk function sets the standard for evidence. Additionally, a downloadable template is available for **budget impact analysis**, ensuring that evidence aligns with the product's function, intended use, and economic impact.	This guide is an update to the 'Code of Conduct for Data-Driven Health and Care Technologies'. We hear from innovators that they valued the practical advice within the Code and this has been strengthened and expanded upon in this update.<br>1. How to operate ethically<br>2. Have a clear value proposition<br>3. Usability and accessibility<br>4. Technical assurance<br>5. Clinical safety<br>6. Data protection<br>7. Data transparency<br>8. Cybersecurity<br>9. Regulation<br>10. Interoperability and open standards<br>11. Generate evidence that the product achieves clinical, social, economic or behavioural benefits<br>12. Define the commercial strategy<br><br>NICE has created the META tool to help companies understand the kind of evidence needed to create a convincing case to commissioners. NICE and others has also developed an Evidence Standards Framework for digital health technologies to inform technology developers and evaluators about which types of evidence should be generated. NICE's technology evaluation programmes, such as Medical Technologies Evaluation Programme (MTEP), consider products that could offer substantial benefits to patients and the health and social care system over current practice.
7.	**US Executive Order on AI** / 2023 [36]	US vows to lead in advancing and governing the development and use of AI for the sake of security, economy, and society. There are eight guiding principles and priorities.	The Executive Order provides further information in Sec. 3 on Definitions, and provides further details on proposed actions to realise each of the principle:<br>• Sec. 4 on Ensuring the Safety and Security of AI Technology: To develop guidelines, standards, and best practices for AI safety and security. To manage AI in

No.	Name/Organisation/Year	Core Value/Principle	Content/Policy
		1. AI must be safe and secure. Effective labelling and content provenance mechanisms to determine when content is generated using AI and when it is not.<br>2. Promoting responsible innovation, competition, and collaboration with investments in AI-related education, training, development, research, and capacity, while simultaneously tackling novel intellectual property questions and other problems to protect inventors and creators. Promote a fair, open, and competitive ecosystem and marketplace for AI and related technologies so that small developers and entrepreneurs can continue to drive innovation.<br>3. AI to improve workers' lives, positively augment human work. AI should not undermine rights, encourage undue worker surveillance, lessen market competition, introduce new health and safety risks, or cause harmful labour-force disruptions.<br>4. AI policies must be advancing equity and civil rights. AI systems should responsibly be deployed to avoid reproducing and intensifying existing inequities, causing new types of harmful discrimination, and exacerbating online and physical harms.<br>5. To enforce existing consumer protection laws and principles and enact appropriate safeguards against fraud, unintended bias, discrimination, infringements on privacy, and other harms from AI.<br>6. To ensure that the collection, use, and retention of data is lawful, is secure, and mitigates privacy and confidentiality risks to protect people's private identities, locations, habits, desires, and to combat the broader legal and societal risks that result from the improper collection and use of people's data.<br>7. To ensure that all members of its own workforce receive adequate training to understand the benefits, risks, and limitations of AI for their job functions, and	critical infrastructure and in cybersecurity. To reduce the risks posed by synthetic content. To solicit input on dual-Use foundation models with widely available model weights. To promote safe release and preventing the malicious use of federal data for AI training.<br>• Sec. 5 on Promoting Innovation and Competition: To attract AI talent to US. To promote innovation and competition in related industries.<br>• Sec. 6 on Supporting Workers: To advance the understanding of AI's implications for workers. To help ensure that AI deployed in the workplace advances employees' well-being. To foster a diverse AI-ready workforce.<br>• Sec. 7 on Advancing Equity and Civil Rights: To strengthen AI and civil rights in the criminal justice system. To protect civil rights related to government benefits and programs. To strengthen AI and civil rights in the broader economy.<br>• Sec. 8 on Protecting Consumers, Patients, Passengers, and Students.<br>• Sec. 9 on Protecting Privacy To advance research, development, and implementation related to PETs.<br>• Sec. 10 on Advancing Federal Government Use of AI and Increasing AI Talent in Government.<br>• Sec. 11 on Strengthening American Leadership Abroad.<br>• Sec. 12 on Implementation.

No.	Name/Organisation/Year	Core Value/Principle	Content/Policy
		to modernise information technology infrastructure, remove bureaucratic obstacles, and ensure that safe and rights-respecting AI is adopted, deployed, and used.<br>8. US aspires to lead the global societal, economic, and technological progress. To pioneer and promote those systems and safeguards needed to deploy technology responsibly with the rest of the world. To engage with international allies and partners in developing a framework to manage AI's risks, unlock AI's potential for good, and promote common approaches to shared challenges. To promote responsible AI safety and security principles and actions with all nations, while leading key global conversations and collaborations to ensure that AI benefits the whole world, rather than exacerbating inequities, threatening human rights, and causing other harms.	
8.	**Artificial Intelligence Act /** European Parliament / 2024 [37]	The purpose of this Regulation is to lay down a uniform legal framework for the development, the placing on the market, the putting into service and the use of non-military (not related to defence or national security purposes) AI systems in the European Union (EU), in accordance with the EU values, to promote the uptake of human-centric and trustworthy AI while ensuring a high level of protection of health, safety, fundamental rights as enshrined in the Charter of the fundamental rights of EU including democracy, the rule of law and environmental protection, and against the harmful effects of AI systems in EU, and to support innovation. This Regulation ensures the free movement, cross- border of AI-based goods and services, thus preventing Member States from imposing restrictions on development, marketing and use of AI systems unless explicitly authorised by this Regulation.<br>The Act lay down:	The Act documents 13 chapters and 13 annexes in 459 pages.<br>CHAPTER I: General Provisions on the subject matters, scopes covered, definitions, and AI literacy.<br>CHAPTER II:<br>Prohibited Artificial Intelligence Practices<br>CHAPTER III: High-Risk AI Systems on classification, requirements, obligations of providers and deployers of high-risk AI systems, and establishing procedure in notifying authorities and notified bodies; standards, conformity assessment, certificates, and registration.<br>CHAPTER IV: Transparency Obligations for Providers and Deployers of Certain AI Systems.<br>CHAPTER V: General-purpose AI Models on classification, requirements, authorised representatives, obligations of providers and deployers of general-purpose AI systems with systemic risk, and codes of practice.<br>CHAPTER VI: Measures In Support of Innovation.

No.	Name/Organisation/Year	Core Value/Principle	Content/Policy
		(a) harmonised rules for the placing on the market, the putting into service, and the use of AI systems in the EU.<br>(b) prohibitions of certain AI practices (see below).<br>(c) specific requirements for high-risk AI systems and obligations for operators of such systems.<br>(d) harmonised transparency rules for certain AI systems.<br>(e) harmonised rules for the placing on the market of general-purpose AI models.<br>(f) rules on market monitoring, market surveillance governance and enforcement.<br>(g) measures to support innovation, with a particular focus on SMEs, including start- ups.<br><br>The seven prohibited AI systems are those that<br>1. deploys subliminal techniques beyond a person's consciousness or purposefully manipulative or using deceptive techniques with the objective of distorting the behaviour of a person/s by impairing their ability to make an informed decision thereby causing a person to take a decision that would not have otherwise taken in a manner that causes or is likely to cause significant harms.<br>2. exploits vulnerable group of persons due to their age, disability or any specific social or economic condition with the objective of distorting the behaviour of that group of persons in a manner that causes or is reasonably likely to cause significant harms.<br>3. evaluate or classify people over a certain period of time based on their social behaviours known, inferred, predicted or personality characteristics with a kind of social score leading to unjustified or disproportionate to their social behaviour, detrimental or unfavourable treatment of the people in social contexts that are unrelated to the contexts in which the data was originally generated or collected.	CHAPTER VII: Governance on governance at EU level, AI Office, advisory forum, scientific panel of independent experts, and access to the pool of experts.<br>CHAPTER VIII: EU Database for High-Risk AI Systems.<br>CHAPTER IX: Post-market Monitoring, Information Sharing, and Market Surveillance on also enforcement of this Act.<br>CHAPTER X: Codes of Conduct and Guidelines.<br>CHAPTER XI: Delegation of Power and Committee Procedure.<br>CHAPTER XII: Penalties.<br>CHAPTER XIII: Final Provisions.<br>ANNEX I: List of Union harmonisation legislation.<br>ANNEX II: List of criminal offences referred to in Article 5(1), point (e)(iii).<br>ANNEX III: High-risk AI systems referred to in Article 6(2).<br>ANNEX IV: Technical documentation referred to in Article 11(1).<br>ANNEX V: EU declaration of conformity.<br>ANNEX VI: Conformity assessment procedure based on internal control.<br>ANNEX VII: Conformity based on an assessment of the quality management system and an assessment of the technical documentation.<br>ANNEX VIII: Information to be submitted upon the registration of high-risk AI systems in accordance with Article 49.<br>ANNEX IX: Information to be submitted upon the registration of high-risk AI systems listed in Annex III in relation to testing in real world conditions in accordance with Article 60.<br>ANNEX X: Union legislative acts on large-scale IT systems in the area of Freedom, Security and Justice.<br>ANNEX XI: Technical documentation referred to in Article 53(1), point (a) - technical documentation for providers of general-purpose AI models.

No.	Name/Organisation/Year	Core Value/Principle	Content/Policy
		4. make risk assessments of natural persons in order to assess or predict the likelihood of a natural person committing a criminal offence based solely on the profiling of their personality traits and characteristics. This prohibition shall not apply to AI systems used to support the human assessment of involvement of a person in a criminal activity, which is already based on objective and verifiable facts directly linked to a criminal activity.<br>5. create or expand facial recognition databases through the untargeted scraping of facial images from the internet or CCTV footage.<br>6. infer emotions of a natural person in the areas of workplace and education institutions, except where the use of the AI system is intended for medical or safety reasons.<br>7. use of biometric categorisation systems that categorise natural persons to deduce or infer their race, political opinions, trade union membership, religious or philosophical beliefs, sex life or sexual orientation. This prohibition does not cover any labelling or filtering of lawfully acquired biometric datasets such as images or biometric data in law enforcement.	ANNEX XII: Transparency information referred to in Article 53(1), point (b)- technical documentation for providers of general-purpose AI models to downstream providers that integrate the model into their AI systems.<br>ANNEX XIII: Criteria for the designation of general-purpose AI models with systemic risk referred to in Article 5.
9.	**Ethics and governance of artificial intelligence for health** /<br>WHO guidance / 2021 [38]	The report provides practical advice for implementing the WHO guidance for three sets of stakeholders: AI technology developers, ministries of health and health-care providers (as a summarised appendix). The considerations are intended only as a starting-point for context-specific discussions and decisions by diverse stakeholders. The primary readership of this guidance document is ministries of health, it is also intended for other government agencies, ministries that will regulate AI, those who use AI technologies for health and entities that design and finance AI technologies for health. The report identifies the ethical challenges and risks with the	This comprehensive document aims to provide guidance into the future on AI uses across healthcare industries from clinical care, research and public health. It contains 9 sections and an extensive subtopic:<br>1. Introduction explains the rationale for WHO's engagement in this topic.<br>2. Artificial intelligence (AI) defined.<br>3. Applications of AI for health provides a non-comprehensive classification and examples of AI technologies for health, medicine,<br>health research, drug development, health systems management and planning, and public health surveillance.

No.	Name/Organisation/Year	Core Value/Principle	Content/Policy
		use of AI of health, six consensus principles to ensure AI works to the public benefit of all countries. It also contains a set of recommendations that can ensure the governance of artificial intelligence for health maximizes the promise of the technology and holds all stakeholders – in the public and private sector – accountable and responsive to the healthcare workers who will rely on these technologies and the communities and individuals whose health will be affected by its use. The six core principles are:<br>1) Protect autonomy<br>2) Promote human well-being, human safety, and the public interest<br>3) Ensure transparency, explainability, and intelligibility<br>4) Foster responsibility and accountability<br>5) Ensure inclusiveness and equity<br>6) Promote AI that is responsive and sustainable.	4. Laws, policies and principles that apply to use of AI for health summarises the laws, policies and principles that apply or could apply to the use of AI for health.<br>5. Key ethical principles for use of AI for health describes six ethical principles that guide the development and use of AI for health.<br>6. Ethical challenges to use of AI for health care presents the ethical<br>challenges to which the guiding ethical principles can be applied.<br>7. Building an ethical approach to use of AI for health examines how various stakeholders can introduce ethical practices,<br>programmes and measures to anticipate or meet ethical norms and legal obligations.<br>8. Liability regimes for AI for health discusses of how liability regimes may evolve with increasing use of AI for health care.<br>9. Elements of a framework for governance of AI for health presents elements of a governance framework for AI for health.
10.	**OECD AI Principles** / 2019[39]	This Recommendation was participatory in development, incorporating input from a broad range of sources throughout the process. The AI Group of experts at the OECD (AIGO) comprising over 50 experts from different disciplines and different sectors (government, industry, civil society, trade unions, the technical community and academia) met from September 2018 to February 2019 before the Recommendation is finally accepted by the OECD Committee on Digital Economy Policy (CDEP) in May 2019. The OECD AI Principles focus on how governments and other actors can shape a human-centric approach to trustworthy AI. As an OECD legal instrument, the principles represent a common aspiration for its adhering countries.<br>Values-based principles of AI systems:	This Recommendation also offers guidance for consideration by policy makers with the purpose of maximizing and sharing the benefits from AI, while minimizing the risks and concerns, with special attention to international cooperation and inclusion of developing countries and underrepresented populations.<br><br>Recommendations for policy makers:<br>1. Investing in AI research and development from public and private investment in research and development to spur innovation in trustworthy AI.<br>2. Fostering a digital ecosystem for AI with infrastructure and technologies, and mechanisms to share data and knowledge.<br>3. Providing an enabling policy environment for AI that will open the way to deployment of trustworthy AI systems.

No.	Name/Organisation/Year	Core Value/Principle	Content/Policy
		9. Inclusive growth, sustainable development and well-being for all individuals, society and planet.<br>10. Human-centred values and fairness that include respecting the rule of law, human rights, democratic values and diversity, and appropriate safeguards to ensure a fair and just society.<br>11. Transparency and explainability are to ensure that people understand when they are engaging with AI systems, and can challenge the outcomes.<br>12. Robustness, security and safety throughout their lifetimes, and potential risks should be continually assessed and managed.<br>13. Accountability of the organisations and individuals who develop, deploy or operate AI systems for their proper functioning in line with the OECD's values-based principles for AI.	4. Building human capacity and preparing for labour market transition<br>5. International co-operation for trustworthy AI that share information, develop standards and work towards responsible stewardship of AI.<br><br>There are definitions for some AI terms and concepts:<br>1. AI system<br>2. AI system lifecycle<br>3. AI knowledge<br>4. AI actors<br>5. Stakeholders<br>6. Trustworthy AI

No.	Name/Organisation/Year	Core Value/Principle	Content/Policy
11.	**Universal Guidelines for AI** / CAIDP / 2018 [40]	The Universal Guidelines on Artificial Intelligence (UGAI) proposes recommendations that can improve and inform AI systems design. The purpose of the UGAI is to promote transparency and accountability for these systems and to ensure that people retain control over the systems they create, and above all else, these systems should do no harm. The 12 principles are:<br>1. Transparency aim to enable independent accountability for automated decisions, with a primary emphasis on the right of the individual to know the basis of an adverse determination.<br>2. Right to a Human Determination reaffirms that individuals and not machines are responsible for automated decision-making, and requires that a human assessment of the outcome to be made.<br>3. Identification Obligation establishes the foundation of AI accountability which is to make clear the identity of an AI system and the institution responsible.<br>4. Fairness Obligation recognizes that all automated systems make decisions that reflect bias and discrimination, but such decisions should not be normatively unfair. Assessment of objective outcomes alone is not sufficient to evaluate an AI system. Normative consequences must be assessed, including those that preexist or may be amplified by an AI system.<br>5. Assessment and Accountability Obligation speaks to the obligation to assess an AI system prior to and during deployment include determining whether an AI system should be established.<br>6. Accuracy, Reliability, and Validity Obligations set out key responsibilities associated with the outcome of automated decisions.<br>7. Data Quality Principle follows from the preceding obligation.	The UGAI builds on prior work by scientific societies, think tanks, NGOs, and international organizations. It incorporates elements of human rights doctrine, data protection law, and ethical guidelines. UGAI is to be incorporated into ethical standards, adopted in national law and international agreements, and built into the design of systems.

No.	Name/Organisation/Year	Core Value/Principle	Content/Policy
		8. Public Safety Obligation recognizes that AI systems control devices in the physical world, and institutions must both assess public risks and take precautionary measures as appropriate.<br>9. Cybersecurity Obligation underscores the risk that even well-designed systems may be the target of hostile actors, and those who develop and deploy AI systems must take these risks into account.<br>10. Prohibition on Secret Profiling aim is to avoid the information asymmetry that arises increasingly with AI systems and to ensure the possibility of independent accountability.<br>11. Prohibition on Unitary Scoring speaks against a single, multi-purpose number assigned by a government to profile an individual's traits or status.<br>12. Termination Obligation presumes that systems must remain within human control. If that is no longer possible, the system should be terminated.	

The G20 AI Principles published in 2019 are drawn from the OECD principles and recommendations.
CAIDP= Center for AI and Digital Policy; OECD= Organization for Economic Cooperation and Development; UNESCO= United Nations Educational, Scientific and Cultural Organization; UN= United Nations; US= United States of America; WHO= World Health Organization

Reference

1. Van Smeden M, Moons C, Hooft L, Kant I, van Os H, Chavannes N. Guideline for high-quality diagnostic and prognostic applications of AI in healthcare. The Netherlands: University Medical Center Utrecht (UMCU) and Leiden University Medical Center (LUMC); 2023 Oct. Report No.: Version 1.1 16-08-2023. Available from: https://doi.org/10.17605/OSF.IO/TNRJZ

2. Kwong JCC, Khondker A, Lajkosz K, McDermott MBA, Frigola XB, McCradden MD, Mamdani M, Kulkarni GS, Johnson AEW. APPRAISE-AI Tool for Quantitative Evaluation of AI Studies for Clinical Decision Support. JAMA Netw Open 2023 Sept 25;6(9):e2335377. doi: 10.1001/jamanetworkopen.2023.35377

3. Kotecha D, Asselbergs FW, Achenbach S, Anker SD, Atar D, Baigent C, Banerjee A, Beger B, Brobert G, Casadei B, Ceccarelli C, Cowie MR, Crea F, Cronin M, Denaxas S, Derix A, Fitzsimons D, Fredriksson M, Gale CP, Gkoutos GV, Goettsch W, Hemingway H, Ingvar M, Jonas A, Kazmierski R, Løgstrup S, Lumbers RT, Lüscher TF, McGreavy P, Piña IL, Roessig L, Steinbeisser C, Sundgren M, Tyl B, Thiel GV, Bochove KV, Vardas PE, Villanueva T, Vrana M, Weber W, Weidinger F, Windecker S, Wood A, Grobbee DE. CODE-EHR best-practice framework for the use of structured electronic health-care records in clinical research. Lancet Digit Health 2022 Oct;4(10):e757–e764. doi: 10.1016/S2589-7500(22)00151-0

4. Vasey B, Nagendran M, Campbell B, Clifton DA, Collins GS, Denaxas S, Denniston AK, Faes L, Geerts B, Ibrahim M, Liu X, Mateen BA, Mathur P, McCradden MD, Morgan L, Ordish J, Rogers C, Saria S, Ting DSW, Watkinson P, Weber W, Wheatstone P, McCulloch P, the DECIDE-AI expert group, Lee AY, Fraser AG, Connell A, Vira A, Esteva A, Althouse AD, Beam AL, De Hond A, Boulesteix A-L, Bradlow A, Ercole A, Paez A, Tsanas A, Kirby B, Glocker B, Velardo C, Park CM, Hehakaya C, Baber C, Paton C, Johner C, Kelly CJ, Vincent CJ, Yau C, McGenity C, Gatsonis C, Faivre-Finn C, Simon C, Sent D, Bzdok D, Treanor D, Wong DC, Steiner DF, Higgins D, Benson D, O'Regan DP, Gunasekaran DV, Danks D, Neri E, Kyrimi E, Schwendicke F, Magrabi F, Ives F, Rademakers FE, Fowler GE, Frau G, Hogg HDJ, Marcus HJ, Chan H-P, Xiang H, McIntyre HF, Harvey H, Kim H, Habli I, Fackler JC, Shaw J, Higham J, Wohlgemut JM, Chong J, Bibault J-E, Cohen JF, Kers J, Morley J, Krois J, Monteiro J, Horovitz J, Fletcher J, Taylor J, Yoon JH, Singh K, Moons KGM, Karpathakis K, Catchpole K, Hood K, Balaskas K, Kamnitsas K, Militello L, Wynants L, Oakden-Rayner L, Lovat LB, Smits LJM, Hinske LC, ElZarrad MK, Van Smeden M, Giavina-Bianchi M, Daley M, Sendak MP, Sujan M, Rovers M, DeCamp M, Woodward M, Komorowski M, Marsden M, Mackintosh M, Abramoff MD, De La Hoz MÁA, Hambidge N, Daly N, Peek N, Redfern O, Ahmad OF, Bossuyt PM, Keane PA, Ferreira PNP, Schnell-Inderst P, Mascagni P, Dasgupta P, Guan P, Barnett R, Kader R, Chopra R, Mann RM, Sarkar R, Mäenpää SM, Finlayson SG, Vollam S, Vollmer SJ, Park SH, Laher S, Joshi S, Van Der Meijden SL, Shelmerdine SC, Tan T-E, Stocker TJW, Giannini V, Madai VI, Newcombe V, Ng WY, Rogers WA, Ogallo W, Park Y, Perkins ZB. Reporting guideline for the early-stage clinical evaluation of decision support systems driven by artificial intelligence: DECIDE-AI. Nat Med 2022 May;28(5):924–933. doi: 10.1038/s41591-022-01772-9

5. Cruz Rivera S, Liu X, Chan A-W, Denniston AK, Calvert MJ, The SPIRIT-AI and CONSORT-AI Working Group, SPIRIT-AI and CONSORT-AI Steering Group, Darzi A, Holmes C, Yau C, Moher D, Ashrafian H, Deeks JJ, Ferrante Di Ruffano L, Faes L, Keane PA, Vollmer SJ, SPIRIT-AI and CONSORT-AI Consensus Group, Lee AY, Jonas A, Esteva A, Beam AL, Panico MB, Lee CS, Haug C, Kelly CJ, Yau C, Mulrow C, Espinoza C, Fletcher J, Moher D, Paltoo D, Manna E, Price G, Collins GS, Harvey H, Matcham J, Monteiro J, ElZarrad MK, Ferrante Di Ruffano L, Oakden-Rayner L, McCradden M, Keane PA, Savage R, Golub R, Sarkar R, Rowley S. Guidelines for clinical trial protocols for interventions involving artificial intelligence: the SPIRIT-AI extension. Nat Med 2020 Sept;26(9):1351–1363. doi: 10.1038/s41591-020-1037-7

6. Liu X, Rivera SC, Moher D, Calvert MJ, Denniston AK. Reporting guidelines for clinical trial reports for interventions involving artificial intelligence: the CONSORT-AI Extension. BMJ 2020 Sept 9;m3164. doi: 10.1136/bmj.m3164

7. Collins GS, Reitsma JB, Altman DG, Moons KGM. Transparent reporting of a multivariable prediction model for individual prognosis or diagnosis (TRIPOD): the TRIPOD statement. BMJ 2015 Jan 7;350(jan07 4):g7594–g7594. doi: 10.1136/bmj.g7594

8. Collins GS, Moons KGM, Dhiman P, Riley RD, Beam AL, Van Calster B, Ghassemi M, Liu X, Reitsma JB, Van Smeden M, Boulesteix A-L, Camaradou JC, Celi LA, Denaxas S, Denniston AK, Glocker B, Golub RM, Harvey H, Heinze G, Hoffman MM, Kengne AP, Lam E, Lee N, Loder EW, Maier-Hein L, Mateen BA, McCradden MD, Oakden-Rayner L, Ordish J, Parnell R, Rose S, Singh K, Wynants L, Logullo P. TRIPOD+AI statement: updated guidance for reporting clinical prediction models that use regression or machine learning methods. BMJ 2024 Apr 16;e078378. doi: 10.1136/bmj-2023-078378

9. Moons KGM, Wolff RF, Riley RD, Whiting PF, Westwood M, Collins GS, Reitsma JB, Kleijnen J, Mallett S. PROBAST: A Tool to Assess Risk of Bias and Applicability of Prediction Model Studies: Explanation and Elaboration. Ann Intern Med 2019 Jan 1;170(1):W1. doi: 10.7326/M18-1377

10. Collins GS, Dhiman P, Andaur Navarro CL, Ma J, Hooft L, Reitsma JB, Logullo P, Beam AL, Peng L, Van Calster B, Van Smeden M, Riley RD, Moons KG. Protocol for development of a reporting guideline (TRIPOD-AI) and risk of bias tool (PROBAST-AI) for diagnostic and prognostic prediction model studies based on artificial intelligence. BMJ Open 2021 July;11(7):e048008. doi: 10.1136/bmjopen-2020-048008

11. Moons KGM, Damen JAA, Kaul T, Hooft L, Andaur Navarro C, Dhiman P, Beam AL, Van Calster B, Celi LA, Denaxas S, Denniston AK, Ghassemi M, Heinze G, Kengne AP, Maier-Hein L, Liu X, Logullo P, McCradden MD, Liu N, Oakden-Rayner L, Singh K, Ting DS, Wynants L, Yang B, Reitsma JB, Riley RD, Collins GS, Van Smeden M. PROBAST+AI: an updated quality, risk of bias, and applicability assessment tool for prediction models using regression or artificial intelligence methods. BMJ 2025 Mar 24;e082505. doi: 10.1136/bmj-2024-082505

12. Sounderajah V, Ashrafian H, Golub RM, Shetty S, De Fauw J, Hooft L, Moons K, Collins G, Moher D, Bossuyt PM, Darzi A, Karthikesalingam A, Denniston AK, Mateen BA, Ting D, Treanor D, King D, Greaves F, Godwin J, Pearson-Stuttard J, Harling L, McInnes M, Rifai N, Tomasev N, Normahani P, Whiting P, Aggarwal R, Vollmer S, Markar SR, Panch T, Liu X. Developing a reporting guideline for artificial intelligence-centred diagnostic test accuracy studies: the STARD-AI protocol. BMJ Open 2021 June;11(6):e047709. doi: 10.1136/bmjopen-2020-047709

13. Sounderajah V, Guni A, Liu X, Collins GS, Karthikesalingam A, Markar SR, Golub RM, Denniston AK, Shetty S, Moher D, Bossuyt PM, Darzi A, Ashrafian H, STARD-AI Steering Committee, Acharya A, Mateen BA, Kelly C, Ting D, Treanor D, King D, Greaves F, Harvey H, De Fauw J, Cohen JF, Godwin J, Pearson-Stuttard J, Moons K, Harling L, Maier-Hein L, Hooft L, McInnes MD, Rifai N, Tomasev N, Normahani P, Whiting P, Aggarwal R, Vollmer S, Markar SR, Panch T, STARD-AI Consensus Group, Ben Glocker, Taylor D, Moher D, Samuel D, Ordish J, Singh K, Celi L, Bossuyt P, Rose S, Saria S. The STARD-AI reporting guideline for diagnostic accuracy studies using artificial intelligence. Nat Med 2025 Sept 15; doi: 10.1038/s41591-025-03953-8

14. Hernandez-Boussard T, Bozkurt S, Ioannidis JPA, Shah NH. MINIMAR (MINimum Information for Medical AI Reporting): Developing reporting standards for artificial intelligence in health care. J Am Med Inform Assoc 2020 Dec 9;27(12):2011–2015. doi: 10.1093/jamia/ocaa088

15. Mongan J, Moy L, Kahn CE. Checklist for Artificial Intelligence in Medical Imaging (CLAIM): A Guide for Authors and Reviewers. Radiol Artif Intell 2020 Mar 1;2(2):e200029. doi: 10.1148/ryai.2020200029

16. Norgeot B, Quer G, Beaulieu-Jones BK, Torkamani A, Dias R, Gianfrancesco M, Arnaout R, Kohane IS, Saria S, Topol E, Obermeyer Z, Yu B, Butte AJ. Minimum information about clinical artificial intelligence modeling: the MI-CLAIM checklist. Nat Med 2020 Sept;26(9):1320–1324. doi: 10.1038/s41591-020-1041-y

17. Hawksworth C, Elvidge J, Knies S, Zemplenyi A, Petykó Z, Siirtola P, Chandra G, Srivastava D, Denniston A, Chalkidou A, Delaye J, Nousios P, Gomes M, Avsar TS, Wang J, Petrou S, Dawoud D. Protocol for the development of an artificial intelligence extension to the Consolidated Health Economic Evaluation Reporting Standards (CHEERS) 2022. 2023. doi: 10.1101/2023.05.31.23290788

18. Elvidge J, Hawksworth C, Avşar TS, Zemplenyi A, Chalkidou A, Petrou S, Petykó Z, Srivastava D, Chandra G, Delaye J, Denniston A, Gomes M, Knies S, Nousios P, Siirtola P, Wang J, Dawoud D. Consolidated Health Economic Evaluation Reporting Standards for Interventions that use Artificial Intelligence (CHEERS-AI). Value Health 2024 May;S1098301524023660. doi: 10.1016/j.jval.2024.05.006

19. Bilbro NA, Hirst A, Paez A, Vasey B, Pufulete M, Sedrakyan A, McCulloch P. The IDEAL Reporting Guidelines: A Delphi Consensus Statement Stage Specific Recommendations for Reporting the Evaluation of Surgical Innovation. Ann Surg 2021 Jan;273(1):82–85. doi: 10.1097/SLA.0000000000004180

20. McCulloch P, Altman DG, Campbell WB, Flum DR, Glasziou P, Marshall JC, Nicholl J. No surgical innovation without evaluation: the IDEAL recommendations. The Lancet 2009 Sept;374(9695):1105–1112. doi: 10.1016/S0140-6736(09)61116-8

21. Marcus HJ, Bennett A, Chari A, Day T, Hirst A, Hughes-Hallett A, Kolias A, Kwasnicki RM, Martin J, Rovers M, Squire SE, McCulloch P. IDEAL-D Framework for Device Innovation: A Consensus Statement on the Preclinical Stage. Ann Surg 2022 Jan;275(1):73–79. doi: 10.1097/SLA.0000000000004907

22. Lekadir K, Osuala R, Gallin C, Lazrak N, Kushibar K, Tsakou G, Aussó S, Alberich LC, Marias K, Tsiknakis M, Colantonio S, Papanikolaou N, Salahuddin Z, Woodruff HC, Lambin P, Martí-Bonmatí L. FUTURE-AI: Guiding Principles and Consensus Recommendations for Trustworthy Artificial Intelligence in Medical Imaging. [object Object]; 2021; doi: 10.48550/ARXIV.2109.09658

23. Fehr J, Citro B, Malpani R, Lippert C, Madai VI. A trustworthy AI reality-check: the lack of transparency of artificial intelligence products in healthcare. Front Digit Health 2024 Feb 20;6:1267290. doi: 10.3389/fdgth.2024.1267290

24. European Commission. Ethics Guidelines for Trustworthy AI. Brussels: European Commission; 2019 Apr. Available from: https://ec.europa.eu/futurium/en/ai-alliance-consultation.1.html [accessed Mar 20, 2024]

25. Liu X, Glocker B, McCradden MM, Ghassemi M, Denniston AK, Oakden-Rayner L. The medical algorithmic audit. Lancet Digit Health 2022 May;4(5):e384–e397. doi: 10.1016/S2589-7500(22)00003-6

26. Kiyasseh D, Cohen A, Jiang C, Altieri N. A framework for evaluating clinical artificial intelligence systems without ground-truth annotations. Nat Commun 2024 Feb 28;15(1):1808. doi: 10.1038/s41467-024-46000-9

27. Callahan A, McElfresh D, Banda JM, Bunney G, Char D, Chen J, Corbin CK, Dash D, Downing NL, Jain SS, Kotecha N, Masterson J, Mello MM, Morse K, Nallan S, Pandya A, Revri A, Sharma A, Sharp C, Thapa R, Wornow M, Youssef A, Pfeffer MA, Shah NH. Standing on FURM ground -- A framework for evaluating Fair, Useful, and Reliable AI Models in healthcare systems. [object Object]; 2024; doi: 10.48550/ARXIV.2403.07911

28. UNESCO. Recommendation on the Ethics of Artificial Intelligence. France: UNESCO; 2022. Report No.: SHS/BIO/PI/2021/1. Available from: https://unesdoc.unesco.org/ark:/48223/pf0000381137.locale=en

29. UNESCO. Readiness assessment methodology. A tool of the Recommendation on the Ethics of Artificial Intelligence. UNESCO; 2023. doi: 10.54678/YHAA4429

30. UNESCO. Ethical impact assessment. A tool of the Recommendation on the Ethics of Artificial Intelligence. UNESCO; 2023. doi: 10.54678/YTSA7796

31. United Nations. Seizing the opportunities of safe, secure and trustworthy artificial intelligence systems for sustainable development. United Nations; 2024 Mar. Report No.: A/78/L.49. Available from: https://documents.un.org/doc/undoc/ltd/n24/065/92/pdf/n2406592.pdf?token=HdJaoM1d02kd1KaU55&fe=true [accessed Apr 22, 2024]

32. Department for Science, Innovation and Technology, AI Safety Institute. International Scientific Report on the Safety of Advanced AI: Interim Report. UK Government; 2024 May. Report No.: DSIT 2024/009. Available from: https://www.gov.uk/government/publications/international-scientific-report-on-the-safety-of-advanced-ai [accessed May 21, 2024]

33. The STANDING Together collaboration. Recommendations for Diversity, Inclusivity, and Generalisability in Artificial Intelligence Health technologies and Health Datasets. Zenodo; 2023. doi: 10.5281/ZENODO.10048356

34. Innovation, Science and Economic Development Canada. Voluntary Code of Conduct on the Responsible Development and Management of Advanced Generative AI Systems. Innov Sci Econ Dev Can. 2023. Available from: https://ised-isde.canada.ca/site/ised/en/voluntary-code-conduct-responsible-development-and-management-advanced-generative-ai-systems [accessed Dec 17, 2024]

35. Department, of Health &, Social Care. A guide to good practice for digital and data-driven health technologies. Digit Data-Driven Health Care Technol. 2021. Available from: https://www.gov.uk/government/publications/code-of-conduct-for-data-driven-health-and-care-technology/initial-code-of-conduct-for-data-driven-health-and-care-technology [accessed Dec 17, 2024]

36. Biden Jr JR. Executive Order on the Safe, Secure, and Trustworthy Development and Use of Artificial Intelligence. 2023. Available from: https://www.whitehouse.gov/briefing-room/presidential-actions/2023/10/30/executive-order-on-the-safe-secure-and-trustworthy-development-and-use-of-artificial-intelligence/ [accessed Apr 22, 2024]

37. European Parliament. Artificial Intelligence Act. The European Parliament; 2024 Mar p. 459. Report No.: P9_TA(2024)0138. Available from: https://www.europarl.europa.eu/doceo/document/TA-9-2024-0138_EN.pdf [accessed Apr 23, 2024]

38. World Health Organization. Ethics and governance of artificial intelligence for health: WHO guidance. Geneva: World Health Organization; 2021. Available from: https://iris.who.int/bitstream/handle/10665/341996/9789240029200-eng.pdf?sequence=1 [accessed Apr 29, 2024]

39. OECD AI. OECD AI Princ Overv. Available from: https://oecd.ai/en/ai-principles [accessed Apr 24, 2024]

40. CAIDP. Center for AI and Digital Policy (CAIDP). Univers Guidel AI. 2018. Available from: https://www.caidp.org/universal-guidelines-for-ai/ [accessed Apr 24, 2024]
