# Supplementary material for: Important Ethical, Technical, and Epidemiological Considerations in an AI Tool Production (ETEPAI): Scoping Review
Source: JMIR AI. 2026 Mar 5;5:e80340. doi: 10.2196/80340 (PMC12977167; doi:10.2196/80340)
Supplement: Multimedia Appendix 2 — Table S3: characteristics of guidelines, checklists, and frameworks related to AI tools (according to the alphabetical and ascending year of the publication). AI: artificial intelligence. [file ai-v5-e80340-s002.pdf]

Table S3: characteristics of guidelines, checklists, and frameworks related to AI tools (according to the alphabetical and ascending year of the publication)

Author Year	Name	Aim	Method	Content	Administration
Luo 2016 [1]	Guidelines for Developing and Reporting Machine Learning Predictive Models in Biomedical Research	To attain a set of reporting guidelines on the use of machine learning predictive models within clinical settings.	Developed through Delphi method of 11 researchers from 3 institutions on 3 different continents.	1) A list of reporting items to be included in a research article for every section of a journal paper from title to discussion on limitation, and<br>2) A set of practical sequential steps for developing predictive models.	Biomedical research community in application of machine learning models and consistent reporting of model specifications and results in research.
Park 2020 [2]	Evaluating artificial intelligence in medicine: phases of clinical research	An evaluation framework for AI in healthcare and methodological considerations for each phase of experimental testing as in the clinical research for drugs and medical devices.	Not clearly reported. Likely to be by the authors themselves.	Phase 0 evaluation contains two parallel efforts: assessment of user needs and development of AI algorithms.<br>Phase 1 involves finding a balance between the benefits and side effects of an intervention.<br>Phase 2 on Efficacy and side effects.<br>phase 3 evaluates efficacy and safety compared to the standard of care through well-designed, large-scale studies.<br>Phase 4 is on safety and effectiveness.	Developers should not evaluate their own tools, and private institutions or dedicated evaluation teams without responsibility for solution development or sales, a commitment to the systematic evaluation by informatics professionals.
Chiang 2021 [3]	Guidelines for Conducting Ethical Artificial	Practical recommendations for understanding and	A systematic search for frameworks to identify ethical implications in study	Three stages of consideration with 15 questions:	AI developers and researchers with an operational set of guidelines

	Intelligence Research in Neurology	addressing ethical ramifications early in methods development stages in neurology research.	design and algorithm choices. Using epilepsy as an example, unintended ethical consequences were explored. Practical recommendations are created based on the above.	Stage 1- Algorithm Conceptualisation<br>Stage 2- Algorithm Development<br>Stage 3- Algorithm Calibration	for conducting ethical AI research and to provide clinicians and peer reviewers with a systematic approach to evaluating the potential ethical consequences of emerging AI research.
Olczak 2021 [4]	CAIR, Clinical AI Research checklist	To provide recommendations and guidelines on reporting AI and ML research to clinicians and other healthcare stakeholders.	Not clearly reported. Likely to be by the authors themselves.	Recommendations for choosing outcome metrics suitable for clinicians. The selected measures are selected for their (1) suitability and (2) their interpretability to a clinician. Guidelines for publishing, reviewing, and evaluating reporting of AI and ML content to clinicians.	Researchers, engineers, clinicians, and other stakeholders can use the checklist to read, present, and evaluate AI research geared towards a healthcare setting.
Reddy 2021 [5]	TEHAI, Translational Evaluation of Healthcare AI framework	To have a practical and comprehensive instrument to assess the translational aspects of AI systems, and evaluation of working clinical systems	A critical review of literature assessed existing evaluation and reporting frameworks and gaps. Using health technology evaluation and translational principles, reporting components were identified, independently reviewed for consensus inclusion in a final framework by an international panel of eight expert.	Three main components: capability, utility and adoption with emphasis on translational and ethical features during the model development and deployment.	Evaluation components can be applied at any stage of the development and deployment of the AI system.
Fasterholdt 2022 [6]	MAS-AI, Model for ASsessing the value of Artificial Intelligence in medical imaging	To have an evidence-based model for assessing the value of AI in medical imaging.	A three-phase process: a literature review of existing AI evaluations in medical imaging, interviews with leading Danish AI	Two steps across nine domains and five process factors. Step 1 covers patient descriptions, AI model development, and initial ethical and legal considerations. Step 2	Support decision making and provide greater transparency for all parties.

			researchers, and two workshops involving decision-makers, patient organizations, and researchers. The model was refined between workshops based on feedback from the multidisciplinary team.	involves a multidisciplinary assessment of the AI application's outcomes in safety, clinical aspects, economics, organizational aspects, and patient aspects.	
Haneef 2022 [7]	Methodological Guidelines	To develop the methodological guidelines to estimate population-based health indicators using linked data and/or ML-techniques with new methods.	Stepwise approach systematically to develop the methodological guidelines: 1) scientific literature review, 2) identification of inspiring examples from European countries and 3) Developing the checklist of guidelines contents.	A checklist of eight items:<br>1. Rationale and objective of the study (i.e., research question)<br>2. Study design<br>3. Linked data sources<br>4. Study population<br>5. Study outcomes<br>6. Data preparation<br>7. Data analysis<br>8. Study limitations	Researchers and epidemiologists to develop and adopt new methods/techniques using linked data and machine learning approaches for their studies.
Tanguay 2022 [8]	RADAR, Radiology AI Deployment and Assessment Rubric	To provide a software evaluation framework to streamline communication with AI software industry and provide healthcare decision makers and radiologists with tools to assess the potential use of AI software	Not clearly reported. Likely to be by the authors themselves.	A systematic approach to the evaluation of a radiology AI product using already established guidelines as is, or further modified and built upon, from specifying and describing clinical radiology AI software through standards for single- or multi-centre pre-deployment evaluation and post-deployment monitoring.	Regulators, healthcare organizations or clinical radiology practices to evaluate AI models before deployment.
Forghani 2023 [9]	A Practical Guide for AI Algorithm Selection for the Radiology Department	Approach for AI software assessment and practical points that should be considered when considering the acquisition and	Not reported. Likely to be by the author.	Four factors to consider:<br>1. Value Proposition: Do I need This AI Tool/App?<br>2. Expected Performance at Your Hospital	Practicing radiologists when selecting an AI algorithm for clinical practice.

		deployment of an AI tool in radiology department.		3. Algorithm trial: Practical Barriers to AI Adoption and intended use<br>4. AI Deployment and Orchestration Platforms	
Kelly 2023 [10]	DATO, Data Algorithm Training Output method	To aid reviewing of clinical radiological literature and research using AI methods to complement evidence-based practise in radiology framework.	Not reported. Likely to be by the authors themselves.	Combining DATO and the Patient intervention comparison outcome (PICO) to access the use of AI to answer a given research question.	Radiologists and related professions to systematically assess AI research and products.
Kocak 2023 [11]	CLEAR, CheckList for EvaluAtion of Radiomics research	To improve the quality and reliability, and the reproducibility of radiomic research	Literature review and co-developed with international experts in radiomics, deep learning, and statistics. A modified Delphi method was utilized in the final selection of the items.	A checklist with 58 items according to the structure of a journal article, online version is available and may be updated here. A shorter version known as CLEAR-S is also available.	Authors and reviewers to use as a documentation standard for radiomic research.
Park 2023 [12]	Radiologist's Guide to Evaluating Publications of Clinical Research on AI	To assist clinical radiologists in critically evaluating different types of clinical research articles involving AI. It did not intend to be a comprehensive checklist or methodological summary for complete clinical evaluation of AI or a reporting guideline.	Not reported. Likely to be by the authors themselves.	Ten key items for readers to check are described, regarding study purpose, function and clinical context of AI, training data, data preprocessing, AI modelling techniques, test data, AI performance, helpfulness and value of AI, interpretability of AI, and code sharing.	Readers to consider when reading publications on AI clinical research.
Bragazzi 2024 [13]	Toward Clinical Generative AI:	To address the concern of incorporation of AI into clinical decision-making	Devised following existing literature review and consultation with experts in	Eleven "verification paradigms," with each paradigm to verify the	A broad range of medical stakeholders including patients, health care

	Conceptual Framework	regarding the reliability and accuracy of AI-generated insights.	the field to ensure that the methodologies were grounded in the academic research and theoretical frameworks with practical insights and recommendations from medical professionals and AI technology specialists.	evidence-based nature of AI in clinical decision-making:<br>1. Quiz, vignette, and knowledge survey<br>2. Historical data comparison<br>3. Expert consensus<br>4. Cross-discipline validation<br>5. Rare or complex simulation and scenario testing<br>6. False myth<br>7. Challenging (or controversial) question<br>8. Real-time monitoring<br>9. Algorithm transparency and audit<br>10. Feedback loop<br>11. Ethical and legal review	professionals, ethicists, and legal experts should be engaged.
Dagan 2024 [14]	OPTICA, Organizational PerspecTIve Checklist for AI solutions adoption	A comprehensive, practical checklist tool to assess AI solutions in health care organizations.	A consensus process involving multiple subject-matter domain experts and decision-makers across the authors' organisation.	Comprises 13 chapters, each containing 3 to 12 checklist items, totalled 77. No scoring but checklist items, that require a qualitative, case-specific evaluation process.	Five main stakeholders to participate in the checklist completion in a predefined and stepwise process<br>1. clinical expert<br>2. AI solution developer (e.g., an industry vendor, academic group, or organizational research and development team)<br>3. organizational data lead group members<br>4. machine learning operations (MLOps) experts

					5. organizational AI lead group members
Gallifant 2025 [15]	TRIPOD-LLM (transparent reporting of a multivariable model for individual prognosis or diagnosis- large language models)	A transparent reporting of a multivariable model for individual prognosis or diagnosis, an extension of the TRIPOD-AI, addressing the unique challenges of LLMs in biomedical applications.	Developed through an expedited Delphi process and expert consensus, TRIPOD-LLM emphasizes transparency, human oversight and task-specific performance reporting.	TRIPOD-LLM provides a comprehensive checklist of 19 main items and 50 subitems, covering key aspects from title to discussion. The guidelines introduce a modular format accommodating various text-only LLM research designs and tasks, with 14 main items and 32 subitems applicable across all categories. There is an interactive website (https://tripod-llm.vercel.app/) facilitating easy guideline completion and PDF generation for submission. This guidance is part of a living document on an interactive website, allowing updates through user feedback, evolving standards, and regular reviews. For the latest recommendations, visit https://tripod-llm.vercel.app/.	It serves as a standardized guide for researchers, journals, healthcare professionals, LLM developers, and institutions in the fast-evolving field of biomedical and healthcare LLMs. It provides essential reporting recommendations for studies on LLM development, tuning, or evaluation. Adopting TRIPOD-LLM ensures clarity, transparency, and accountability, helping administrators appraise study quality, prevent overinterpretation, support reproducibility, and streamline LLM implementation.

References

1. Luo W, Phung D, Tran T, Gupta S, Rana S, Karmakar C, Shilton A, Yearwood J, Dimitrova N, Ho TB, Venkatesh S, Berk M. Guidelines for Developing and Reporting Machine Learning Predictive Models in Biomedical Research: A Multidisciplinary View. J Med Internet Res 2016 Dec 16;18(12):e323. doi: 10.2196/jmir.5870

2. Park Y, Jackson GP, Foreman MA, Gruen D, Hu J, Das AK. Evaluating artificial intelligence in medicine: phases of clinical research. JAMIA Open 2020 Oct 1;3(3):326–331. doi: 10.1093/jamiaopen/ooaa033

3. Chiang S, Picard RW, Chiong W, Moss R, Worrell GA, Rao VR, Goldenholz DM. Guidelines for Conducting Ethical Artificial Intelligence Research in Neurology: A Systematic Approach for Clinicians and Researchers. Neurology 2021 Sept 28;97(13):632–640. doi: 10.1212/WNL.0000000000012570

4. Olczak J, Pavlopoulos J, Prijs J, Ijpma FFA, Doornberg JN, Lundström C, Hedlund J, Gordon M. Presenting artificial intelligence, deep learning, and machine learning studies to clinicians and healthcare stakeholders: an introductory reference with a guideline and a Clinical AI Research (CAIR) checklist proposal. Acta Orthopaedica 2021 Sept 3;92(5):513–525. doi: 10.1080/17453674.2021.1918389

5. Reddy S, Rogers W, Makinen V-P, Coiera E, Brown P, Wenzel M, Weicken E, Ansari S, Mathur P, Casey A, Kelly B. Evaluation framework to guide implementation of AI systems into healthcare settings. BMJ Health Care Inform 2021 Oct;28(1):e100444. doi: 10.1136/bmjhci-2021-100444

6. Fasterholdt I, Kjølhede T, Naghavi-Behzad M, Schmidt T, Rautalammi QTS, Hildebrandt MG, Gerdes A, Barkler A, Kidholm K, Rac VE, Rasmussen BSB. Model for ASsessing the value of Artificial Intelligence in medical imaging (MAS-AI). Int J Technol Assess Health Care 2022;38(1):e74. doi: 10.1017/S0266462322000551

7. Haneef R, Tijhuis M, Thiébaut R, Májek O, Pristaš I, Tolonen H, Gallay A. Methodological guidelines to estimate population-based health indicators using linked data and/or machine learning techniques. Arch Public Health 2022 Dec;80(1):9. doi: 10.1186/s13690-021-00770-6

8. Tanguay W, Acar P, Fine B, Abdolell M, Gong B, Cadrin-Chênevert A, Chartrand-Lefebvre C, Chalaoui J, Gorgos A, Chin AS-L, Prénovault J, Guilbert F, Létourneau-Guillon L, Chong J, Tang A. Assessment of Radiology Artificial Intelligence Software: A Validation and Evaluation Framework. Can Assoc Radiol J 2023 May;74(2):326–333. doi: 10.1177/08465371221135760

9. Forghani R. A Practical Guide for AI Algorithm Selection for the Radiology Department. Seminars in Roentgenology 2023 Apr;58(2):208–213. doi: 10.1053/j.ro.2023.02.006

10. Kelly BS, Judge C, Hoare S, Colleran G, Lawlor A, Killeen RP. How to apply evidence-based practice to the use of artificial intelligence in radiology (EBRAI) using the data algorithm training output (DATO) method. The British Journal of Radiology 2023 Oct 1;96(1150):20220215. doi: 10.1259/bjr.20220215

11. Kocak B, Baessler B, Bakas S, Cuocolo R, Fedorov A, Maier-Hein L, Mercaldo N, Müller H, Orlhac F, Pinto Dos Santos D, Stanzione A, Ugga L, Zwanenburg A. CheckList for EvaluAtion of Radiomics research (CLEAR): a step-by-step reporting guideline for authors and reviewers endorsed by ESR and EuSoMII. Insights Imaging 2023 May 4;14(1):75. doi: 10.1186/s13244-023-01415-8

12. Park SH, Sul A-R, Ko Y, Jang HY, Lee J-G. Radiologist's Guide to Evaluating Publications of Clinical Research on AI: How We Do It. Radiology 2023 Sept 1;308(3):e230288. doi: 10.1148/radiol.230288

13. Bragazzi NL, Garbarino S. Toward Clinical Generative AI: Conceptual Framework. JMIR AI 2024 June 7;3:e55957. doi: 10.2196/55957

14. Dagan N, Devons-Sberro S, Paz Z, Zoller L, Sommer A, Shaham G, Shahar N, Ohana R, Weinstein O, Netzer D, Kotler A, Balicer RD. Evaluation of AI Solutions in Health Care Organizations — The OPTICA Tool. NEJM AI 2024 Aug 22;1(9). doi: 10.1056/AIcs2300269

15. Gallifant J, Afshar M, Ameen S, Aphinyanaphongs Y, Chen S, Cacciamani G, Demner-Fushman D, Dligach D, Daneshjou R, Fernandes C, Hansen LH, Landman A, Lehmann L, McCoy LG, Miller T, Moreno A, Munch N, Restrepo D, Savova G, Umeton R, Gichoya JW, Collins GS, Moons KGM, Celi LA, Bitterman DS. The TRIPOD-LLM reporting guideline for studies using large language models. Nat Med 2025 Jan 8; doi: 10.1038/s41591-024-03425-5
