# Supplementary material for: Important Ethical, Technical, and Epidemiological Considerations in an AI Tool Production (ETEPAI): Scoping Review
Source: JMIR AI. 2026 Mar 5;5:e80340. doi: 10.2196/80340 (PMC12977167; doi:10.2196/80340)
Supplement: Multimedia Appendix 3 — Outline of an AI research proposal. AI: artificial intelligence. [file ai-v5-e80340-s003.pdf]

No.	Outline of an AI Research Proposal
1.	**Topic**<br>The title should be concise and informative about the research question. Avoid use of extraneous words such as "study," "investigation," etc.
2.	**Abstract**<br>A summary of the proposed project.
3.	**Introduction | Literature review**<br>a. Comprehensive understanding of the subject and background.<br>b. Cite and discuss systematic reviews that have been reported on the topic, otherwise conduct a ‘mini’ or a sufficient literature review.<br>c. Know what has been published, and alternatives to the proposed AI tool.<br>d. What issues or controversies remain, reasons for the proposed AI tool and project.<br>e. Present the conceptual framework of variables on the outcome of the study.
4.	**Problem statement**<br>a. Show concise understanding and the important of the research question to answer with testable hypothesis.<br>b. State the expected outcomes and benefits; results that have potential impact to the current care practice.
5.	**Objectives**<br>Clear and measurable general and specific objectives
6.	**Theoretical design**<br>a. Focused research question with clearly conceptualised and operationalised measurable variables, using the PE/ICOTS framework (Population, Exposure, Comparison, Outcome, Timing, Setting).<br>b. Feasible, important and interesting, novel, ethical and relevant of the AI tool/system/application.<br>c. Overall study design: diagnostic test study, prognostic study, cohort, case-control, randomised controlled trial, etc.<br>d. Inclusion and exclusion criteria for participants or in the included datasets.<br>e. Clearly defined the settings of the data sources, and to what setting the tool is to be applied.<br>f. Valid and reliable measurement or measuring tools.<br>g. Define the time points of the study’s time-sensitive variables.<br>h. Deployment strategy: Outline the process for integrating the AI tool into clinical practice, including steps for installation, configuration, and initial testing in the real-world setting.<br>i. Training and support: Provide details on training programs for clinical and IT staff to ensure effective use of the AI tool.<br>j. Monitoring and maintenance: describe the monitoring framework for tracking the AI tool's performance and safety post-deployment. Include plans for regular updates and addressing any identified issues.
7.	**Data collection design**<br>a. Identify all data sources, and how they will be handled.<br>b. Appropriate sampling method (representativeness/comparability) - highest possible validity, lowest risk of biases with respect to available resources and existing constraints.<br>c. Define the ground truth of interest, conditions and outcome events, and rationale (if alternatives exist). Describe the measurement process, and how the data are encoded.<br>d. Outcomes assessor involvement- trained and blinding, if necessary<br>e. Study flow chart to depict the sampling process.
8.	**Data analyses design**<br>a. Sample size estimation, provide the rationale.<br>b. Describe how data are pre-processed, missing data, outliers, noise, and inconsistencies are handled.<br>c. Describe the baseline model and software libraries to be used.<br>d. Model specification: appropriate data splitting for training and testing, features (variables) engineering, choice of model architecture and algorithm selection, statistical tests, hyperparameter tuning and optimisation.<br>e. Model evaluation: specify metrics for model evaluation, such as accuracy, precision, recall, F1 score, and ROC-AUC. Detail methods for internal and external validation.<br>f. To justify decision of sub-group analysis or sensitive analyses,<br>g. Bias and error analysis: include techniques for bias assessment and correction, strategies for error analysis and mitigation, and process audits.<br>h. Dummy tables including the contingency table.

9.	**Discussion**<br>a. Highlight the relevance and importance of the proposed project, research topic or research question.<br>b. Point out the efforts and strength of the project where validity is maximized.<br>c. Show anticipatory thoughtfulness of possible shortfalls and limitations. Shortfalls are the potential threats and biases but indicate efforts to mitigate them. Limitations are imperfections of valid and rigorous study designs for the research question and study objectives, and feasibly planned for the actual conducts, which must be categorically correct but inadequate as required at higher levels. They are not excuses for wrong methods, not confessions of slackness nor explanation of constrained resources as required for the study. These honest declarations are expected once the study is completed.
10.	**Conclusion**<br>Reiterate the importance and feasible of the proposed project, and highest possible validity and precision of the proposed methodology.
11.	**Acknowledgement**<br>Record sources of contributors or contribution that have directly or indirectly made the proposed project possible.
12.	**References**<br>Include all relevant references and literature cited in the proposal.
13.	**Budget**<br>a. Outline a reasonable expenditure to be expected.<br>b. Identify necessary resources, including personnel, computational resources, and data.<br>c. Provide a detailed budget breakdown for the project with brief explanation and supported by recent cost estimation.
14.	**Gantt Chart**<br>Outline the project timeline with important milestones of the project from beginning to the end.
15.	**Supplementary Materials**<br>Include any additional materials such as letters of intent to collaborate or access to critical infrastructures, checklists, detailed methodologies, or supplementary data.
