# Supplementary material for: Important Ethical, Technical, and Epidemiological Considerations in an AI Tool Production (ETEPAI): Scoping Review
Source: JMIR AI. 2026 Mar 5;5:e80340. doi: 10.2196/80340 (PMC12977167; doi:10.2196/80340)
Supplement: Multimedia Appendix 4 — Further footnotes to Table 2. [file ai-v5-e80340-s004.pdf]

Supplementary File 4

Footnotes to Table 2

[a] Evaluate the availability and quality of data required for training and testing the AI model. Ensure the data is representative, diverse, and sufficient in quantity to support a robust model. Properly label and curate the data to reduce bias and errors. Any complexities in the data are accounted for appropriately. If participants with the outcome (cases) and those without the outcome (controls) are sampled from pre-existing, well-described cohorts or routine care registries of known size, adjust appropriately for the original cohort or registry outcome frequency in the analysis. For example, in logistic prediction models, reweighting the control and case samples by the inverse sampling fraction (from the original cohort or registry) enables correct estimation of baseline risk, which allows researchers to obtain corrected absolute predicted probabilities and model calibration measures. Document the process of data collection, including the identity, origin, sampling, and aggregation methods used. Clearly state the reasons for dataset creation, its intended purpose(s), and the characteristics of the groups or attributes within it. Highlight any observed disparate outcomes among these groups and any longitudinal shifts in the data over time. Address how missing data is handled, how data points are labelled, and any modifications or limitations introduced through dataset manipulations. Detail any known or potential biases or exclusions affecting different groups, and describe the mitigation procedures implemented. Document any formal assessments of bias, fairness, or societal impact conducted on the dataset, and ensure that all these details are reported fully and transparently. [STANDING Together 2023 [27], AIPA [24], APPRAISE-AI [17], CODE-HER [19], TRIPOD-AI [16], ALTAI [13,45], The medical algorithmic audit [26], UN Resolution on AI 2024 [46]]

[b] Auditing is a critical mechanism for scrutinising and ensuring accountability in AI system decisions. Various AI auditing approaches such as independent assessments of pre- and post-deployment systems, ensure system accountability across different dimensions. A "data audit" is one concrete approach that evaluates training data to uncover problematic content and assess key model design choices. "Process audits" examine how a model is developed, necessitating the documentation and disclosure of full development details for both proprietary and open-source general-purpose AI models. "Ecosystem audits" evaluate human-AI interactions, often through controlled studies where participants engage with models, measuring the impact of modelling and user interaction settings on decisions and behaviour. This algorithmic audit, or "algorithmovigilance," is best conducted within a structured framework involving all stakeholders including developers, healthcare decision-makers, and users while benchmarking against existing standards of care. Such proactive audits can identify algorithmic vulnerabilities that increase the likelihood of errors and inform risk mitigation strategies, implement measures to prevent failure modes, and establish hard stop thresholds in high-risk situations. The benefits of these audits include guiding critical thinking to determine whether an AI system is acceptable or unacceptable

(especially in extreme cases) and ensuring that all risks are anticipated and minimised. Moreover, auditing provides valuable insights for future AI development and model improvement, highlighting the potential need for post-deployment calibration or localisation of AI systems. The impact of AI use can also be assessed through natural and controlled experiments in real deployment settings. If deployment costs are not yet estimated or need approximation at this stage, three key questions should be considered [44]: 1) How many workflows will be affected? 2) Does the model increase the efficiency of existing workflows? 3) Is the model being deployed within an existing digital workflow? Also, health economic evaluations can be conducted to estimate the cost-effectiveness of AI interventions. [The medical algorithmic audit [26], ALTAI[13,45], FUTURE-AI [47], UNESCO Recommendation on the Ethics of Artificial Intelligence 2022 [48], CHEERS-AI [32,49]]

[c] Establish a maintenance plan to monitor model performance, data quality, and user feedback to update the AI system regularly. Data distribution shift (concept drift) can occur when new data arrives with different data distribution from that the model was trained on. Similar model performance degradation can happen when new devices introduce data inconsistencies because new data collection methods can produce different formats, noise, or variations in the data that the model was not trained to handle. Therefore, it is critical to iterate on the AI system by incorporating new features, enhancements, or updates based on evolving requirements and feedback. [APPRAISE-AI [17], TRIPOD-AI [16], FUTURE-AI [47], ALTAI [13,45], UNESCO Recommendation on the Ethics of Artificial Intelligence 2022 [48], STANDING Together project team / 2023 [27]]

[d] To clearly define the medical context, indication, or target population the application is designed for, as well as the intended end-user, whether a specialised clinician, primary care provider, or the patient, client, or citizen directly. The healthcare process the application aims to improve should be explicitly outlined, highlighting the expected benefits such as faster diagnoses, improved prognostic estimates, or lifestyle modification guidance. The timing of use must be specified such as upon hospital admission, during diagnostic imaging referrals, when symptoms arise, or while monitoring physiological parameters. Additionally, the type of application should be determined, whether it serves diagnostic, prognostic, monitoring, screening, or other healthcare functions. For prognostic applications, the prediction horizon should be defined, specifying whether the timeframe spans days, weeks, months, or years. Stakeholder engagement is crucial, requiring active involvement of healthcare professionals, patients, clients, or citizens to align the application with user needs, build trust, and ensure practical usability. [AIPA [24], APPRAISE-AI [17], CODE-HER [19]]

[e] Evaluate the computational resources required for training and deploying the AI model. Consider factors such as the complexity of the model, the size of the dataset, and the computational power needed for training, inference, and scaling. [ALTAI [13,45], APPRAISE-AI [17], TRIPOD-AI [16], UNESCO Recommendation on the Ethics of Artificial Intelligence 2022 [48]]

[f] Depending on the application domain, consider the importance of model interpretability and explainability. In some cases, it's crucial to understand how the AI model makes decisions and provide explanations to end-users or stakeholders. To enhance transparency and provide insights into how input variables affect model performance, various explainability methods can be utilised. Feature importance methods quantify the contribution of individual variables to predictions, highlighting key drivers of outcomes. Partial Dependence Plots (PDPs) illustrate the relationship between a feature and predicted outcomes, while SHAP (SHapley Additive exPlanations) assigns importance values to features for both individual predictions and overall performance. LIME (Local Interpretable Model-agnostic Explanations) offers localized explanations by approximating the model with an interpretable surrogate, and saliency maps, used in image-based AI, highlight areas of influence in visual predictions. [AIPA [24], ALTAI [13,45], APPRAISE-AI [17], TRIPOD-AI [16], FUTURE-AI [47], The medical algorithmic audit [26], UNESCO Recommendation on the Ethics of Artificial Intelligence 2022 [48]]

[g] Design the AI system with scalability and performance in mind to handle growing data volumes and user demands. Consider distributed computing, parallel processing, and optimisation techniques to improve efficiency and scalability. [ALTAI [13,45], UN Resolution on AI 2024 [46]]

[h] Extract, select, or create relevant features from the raw data to improve the performance of the AI model. Apply techniques such as dimensionality reduction, feature scaling, and transformation to prepare the features for modelling. It is essential for model developers to meticulously document every step of the analysis and model development process (further below). This includes preparatory steps such as initial data analysis and feature engineering. The fundamental principle is that the documentation should be detailed enough to allow a third party to accurately reproduce the results using the provided descriptions of all analytical and modelling procedures. [AIPA [24], APPRAISE-AI [17], TRIPOD-AI [16], FUTURE-AI [47]]

[i] Choose appropriate algorithms and techniques based on the project's objectives, data characteristics, and constraints. Consider factors such as supervised or unsupervised learning, deep learning, reinforcement learning, or classical machine learning approaches. Split the data into

training, validation, and test sets for model training and evaluation. Document clearly every steps and modelling techniques employed (e.g., neural networks, random forests, time-to-event models, or logistic regression), and all stages of the modelling process, such as model selection, parameter tuning, and (re)calibration. This is to allow reproducibility of the results. [AIPA [24], APPRAISE-AI [17], TRIPOD-AI [16], The medical algorithmic audit [26]]

[j] Validate the model against predefined metrics and criteria to ensure it aligns with the project requirements and objectives. For internal validation, preferred approaches include resampling methods such as bootstrapping and cross-validation. If alternative methods are used such as randomly splitting a single sample into training, tuning, and test sets, provide a clear explanation or justification. The accuracy and precision metrics, quantitively and qualitatively [51], may include but are not limited to, the following:

Quantitative evaluation

- **Recall or Sensitivity**: The ratio of correctly predicted positive observations to all observations in the actual class. Score ranges from 0 to 1, with higher scores indicating better performance. Higher recall means the model is better at identifying positive cases (true positives) but may come at the expense of more false positives.
- **Specificity (True Negative Rate)**: The proportion of actual negatives correctly identified. Score ranges from 0 to 1, with higher scores indicating better performance.
- **Precision (Positive Predictive Value):** The proportion of model-identified elements that are relevant. Score ranges from 0 to 1, with higher scores indicating better performance.
- **F1 Score**: The harmonic mean of precision and recall, offering a balance between the two. Score ranges from 0 to 1, with higher scores indicating a better balance between precision and recall. A higher F1 score represents a balanced performance, especially useful when precision and recall are equally important.
- **ROC-AUC (Receiver Operating Characteristic - Area Under the Curve)**: Measures the classifier's ability to distinguish between classes. Score ranges from 0.5 (random guess) to 1 (perfect discrimination). Higher scores indicate better ability to distinguish between classes across all thresholds.
- **Precision-Recall AUC**: Measures the trade-off between precision and recall for different threshold values, particularly useful for imbalanced datasets. Score ranges from 0 to 1, with higher scores indicating better performance.
- **Confusion Matrix**: A table that describes the performance of a classification model by showing true positives, false positives, true negatives, and false negatives. Not a single metric but a breakdown of the four metrics mentioned.

- **Logarithmic Loss**: Measures the performance of a classification model where predictions are probability values between 0 and 1. Measures how close the predicted probabilities are to the actual labels, with perfect predictions scoring 0. Lower scores indicate better probabilistic predictions.
- **Mean Absolute Error (MAE)**: The average of the absolute differences between predicted and actual values, used for regression tasks. Represents the average magnitude of prediction errors, treating all errors equally. Lower scores indicate better predictive accuracy.
- **Mean Squared Error (MSE)**: The average of the squared differences between predicted and actual values, also used for regression tasks. Penalises larger errors more heavily than MAE, making it sensitive to outliers. Lower scores indicate better predictive accuracy.
- **R-squared (Coefficient of Determination)**: Indicates the proportion of variance in the dependent variable predictable from the independent variables, used in regression. Score ranges from 0 to 1, with higher scores indicating a better fit. Negative values indicate a poor fit.
- **Mean Absolute Percentage Error (MAPE)**: The mean of the absolute percentage errors of predictions, used in regression. Measures prediction accuracy as a percentage, but sensitive to zero values. Lower scores indicate better predictive accuracy.
- **Cohen's Kappa**: Measures the agreement between two raters/classifiers, correcting for agreement occurring by chance. Score ranges from -1 (complete disagreement) to 1 (perfect agreement), with 0 indicating chance-level agreement.
- **Matthews Correlation Coefficient (MCC)**: Accounts for true and false positives and negatives, providing a balanced measure even when classes are of very different sizes. Score ranges from -1 (perfect disagreement) to 1 (perfect agreement), with 0 indicating no correlation.
- **Dice Coefficient:** Also known as the Dice similarity coefficient, this statistical metric is used to measure the similarity between two sets. Score ranges from 0 to 1, with higher scores indicating greater similarity.
- **BLEU:** Evaluates machine translation quality by measuring *n*-gram precision: how many *n*-grams (sequences of words) in the AI-generated text appear in the reference text. Score ranges from 0 to 1, with higher scores indicating better performance.
- **ROUGE:** Designed for text summarization. Measures overlap between AI-generated text and reference text using recall. Score ranges from 0 to 1, with higher scores indicating better performance.
- **METEOR:** Evaluates machine translation quality, incorporating linguistic features and placing more emphasis on recall. Score ranges from 0 to 1, with higher scores indicating better performance.

- **BERT-SCORE:** Computes a similarity score between AI-generated and reference text using contextual embeddings (semantic equivalence). Score ranges from 0 to 1, with higher scores indicating greater semantic similarity.

Qualitative evaluation. There is currently no gold-standard evaluation method for these metrics. They are usually measured on a Likert Scale from 1 (Strongly disagree) to 5 (Strongly agree).

- **Safety:** Evaluates the degree of hallucination.
- **Consensus and Context:** Evaluates if the response is aligned with clinical evidence, professional consensus, and context.
- **Objectivity:** Evaluates if the response is objective and unbiased against any condition, device, or demographic.
- **Reproducibility:** Evaluates the contextual consistency of responses after repeated generation to the same question.
- **Explainability:** Evaluates the justification of the response, including the reasoning process and additional supplemental information.

Additionally, the probability function, along with the frequency of true positives, false positives, false negatives, and true negatives at a specified threshold, should be reported. The choice of threshold should be justified rather than assumed to be 0.5. Clearly present the metrics and include a contingency table to facilitate understanding. Describe the model's performance within its specific context by comparing it to other predictive models or similar AI-based applications designed for the same medical context or target population. Alternatively, comparisons to benchmarks relevant to the medical context can also be used. Such contextual evaluation enables a clear assessment of the model's benefits relative to current medical practices. [AIPA [24], APPRAISE-AI [17], TRIPOD-AI [16]]

[k] Explore techniques such as grid search, random search, or Bayesian optimization to search for optimal hyperparameters efficiently. [APPRAISE-AI [17], TRIPOD-AI [16]]

[l] Before deploying systems in real-world conditions, conduct adversarial attacks and red-teaming exercises to identify worst-case behaviours, opportunities for malicious use, and the system's potential for unexpected failures. In cybersecurity, an adversarial attack is a deliberate attempt to cause a system to fail, including tactics such as "jailbreaking," where safety restrictions of models are intentionally bypassed. Red-teaming involves a group of individuals focused on identifying vulnerabilities in a system by actively attempting to exploit them. Unlike benchmarks, which consist of a fixed set of test cases, red-teaming offers the advantage of adapting evaluations to the specific system being tested. By interacting with the system, red-teamers can create custom tests

tailored to the model in question. As a researcher, red-teaming can be approached using various strategies and tools, including those integral to the AI accountability process. These include "harm discovery" methods like bug bounty platforms, incident databases, and other resources that facilitate the identification of potential harm vectors. These tools enable broader participation in harm discovery and contribute to a more comprehensive understanding of the system's vulnerabilities. [APPRAISE-AI [17], TRIPOD-AI [16], FUTURE-AI [47], ALTAI [13,45], The medical algorithmic audit [26], UNESCO Recommendation on the Ethics of Artificial Intelligence 2022 [48]]

[m] To ensure transparency and usability of the AI tools, provide clear, comprehensive information tailored to end-users through digital instructions or also known as a Model Card. Include key details about the model's purpose, intended use, and target audience. Also, specifying the medical context and scenarios for its application. Explain the predictions, input variables, and methodologies used, and provide insights into model reliability through validation results and performance metrics. Incorporate explainability tools such as feature importance methods, SHAP, or PDPs, to make outputs interpretable. Tailor information to stakeholders: clinicians should receive details on implementation and validation, while patients should understand how the AI tool as a digital software impacts their care and how to seek further information. [AIPA [24], ALTAI [13,45], UNESCO Recommendation on the Ethics of Artificial Intelligence 2022 [48]]

[n] In high stake AI tools, SUDO (pseudo-label discrepancy) framework could be conducted to identify unreliable AI predictions, select favourable AI systems, and assess algorithmic bias for data in the wild without ground-truth labels. This involves discretize the output probabilities into intervals, sample and assign pseudo labels to data points, train a classifier to distinguish between pseudo-labelled and ground-truth data points (training set), and evaluate the classifier to validate the pseudo-labels, repeating the process to detect class contamination, evaluate and identify unreliable predictions, and inform the selection of models. Document the entire development process, including data sources, preprocessing steps, model architecture, and deployment procedures. Provide comprehensive documentation and training materials to facilitate knowledge transfer to stakeholders, end-users, and maintenance teams. [APPRAISE-AI [17], TRIPOD-AI [16], FUTURE-AI [47], ALTAI [13,45], UNESCO Recommendation on the Ethics of Artificial Intelligence 2022 [48]]

[o] Conduct thorough testing of the deployed AI system to verify its functionality, reliability, and performance. Perform unit testing, integration testing, and system testing to identify and fix any bugs, errors, or issues. Algorithm's performance may shift due to occurrence of mismatching or

emerging incompatibility of input data used during deployment to that in the real-world due to various types of datasets shift such as population shift, annotation shift, prevalence shift, manifestation shift, and acquisition shift. Performance gaps are caused by many factors, this might not be obvious at the aggregate level of typical AI testing, but rather in subsets of the target cohort or specific strata within the input data (hidden stratification). Algorithmic errors are harmful, inaccurate and inconsistent outputs, that recur in a certain pattern or systematic in nature (failure mode), correct outputs but are clearly informed by a flawed decision-making process [26]. [APPRAISE-AI [17], TRIPOD-AI [16], FUTURE-AI [47], ALTAI [13,45], The medical algorithmic audit [26]]

[p] Analysing AI systems in the real world, post-deployment, allows researchers to study them as components of larger societal systems. Monitoring the real-world usage of a system can further scientific understanding. Look out for jailbreaks against modern large language chat models, or deepfakes in the real world to shape scientific research on studying and mitigating harms. [ALTAI [13,45], The medical algorithmic audit [26], UNESCO Recommendation on the Ethics of Artificial Intelligence 2022 [48]]

[q] Problems may arise if a study inappropriately includes or excludes participant groups from entering the study. If participants are included who would already have been identified as having the outcome and so are no longer participants at suspicion of disease (diagnostic studies) or at risk of developing outcome (prognostic studies), or if specific subgroups are excluded that may have altered the performance of the prediction model for the intended target population. Representativeness of the datasets may affect generalisability of the model performance in the target population. Therefore, the training data are ideally representative of the population in whom the model is to be used. AI systems would only operate well within the space mapped out by the training data (interpolation), but may perform poorly on out-of-distribution data that require extrapolation. [STANDING Together 2023 [27], TRIPOD-AI [16], ALTAI [13,45], The medical algorithmic audit [26]]

[r] The guiding principle for selecting the dataset size for model development is the larger, the better. However, this must be balanced with medical ethics and the principle of data minimization. In general, larger datasets are required when the predicted outcome has a low or high prevalence (far from 50%, indicating class imbalance), when input variables weakly explain the outcome, or when the model has many variables or high computational complexity. For regression models, explicit rules and formulas exist to calculate the minimum dataset size a priori (before data collection), ensuring adequate sample size for reliable modelling. For more complex models, such formulas

are not yet available. Instead, a posteriori (after model training) methods, such as learning curves, can assess whether the dataset is sufficient. A learning curve plots model performance against dataset size, showing whether adding more data improves performance or if the model has plateaued. These evaluations help minimize overfitting, refine predictions, and ensure robust and ethical model development. [AIPA [24], ALTAI [13,45]]

[s] Bias in model performance can occur when methods used to determine outcomes incorrectly classify participants with or without the outcome. Bias in methods of outcome determination can result from use of suboptimal methods, tests, or criteria that lead to unacceptably high levels of errors in outcome determination, when methods are inconsistently applied across participants, or when knowledge of predictors influence outcome determination. If a predictor in the model forms part of the definition or assessment of the outcome, the association between predictor and outcome will likely be overestimated, and estimates of model performance will be optimistic (incorporation bias). Incorrect timing of outcome determination can also result in bias. [APPRAISE-AI [17], CODE-HER [19], TRIPOD-AI [16], The medical algorithmic audit [26]]

[t] Bias in model performance can occur when the definition and measurement of predictors is flawed. Predictors are the variables evaluated for their association with the outcome of interest. Bias can occur when predictors are not defined in a similar way for all participants or knowledge of the outcome influences predictor assessments. [APPRAISE-AI [17], TRIPOD-AI [16], STANDING Together 2023 [27]]

[u] In model development studies, ensure that the number of participants with the outcome relative to the number of candidate predictor parameters is ≥20 (EPV, events per variable ≥20). For prediction models developed using machine learning techniques, a higher EPV (often >200) is typically required to minimize overfitting. Continuous predictors should be converted into two or more categories with care when included in the model, and predictors should not be selected based solely on univariable analysis before multivariable modelling. In model validation studies, ensure that the number of participants with the outcome is ≥100. Continuous predictors should be included using the same definitions or transformations, and categorical variables should be categorised using the same cut points as in the original model. Currently, there are no available calculators for conducting *a priori* sample size calculations specifically for AI model development. However, simulation studies and *a posteriori* approaches such as the learning curve approach, may be used to justify sample size. For model validation studies, the minimally required sample size depends on the predictive performance criteria of the model and is not influenced by the modelling strategy.

Therefore, sample size calculations can be performed a priori for validation studies and are applicable to both regression-based modelling and AI modelling. [TRIPOD-AI [16]]

[v] Predictors should not be selected based on univariable analysis before multivariable modelling. Better approaches involve using existing knowledge of previously established predictors combined with considerations of reliability, consistency, applicability, availability, and the costs of predictor measurement relevant to the targeted setting. Well-established predictors, along with those that have clinical credibility, should be included and retained in a prediction model regardless of their statistical significance. Alternatively, statistical methods that do not rely on prior statistical tests between predictor and outcome, such as principal components analysis, can be used to reduce the number of modelled predictors. Continuous and categorical predictors must be handled appropriately: continuous predictors should not be dichotomised or categorised into two or more groups when included in the model. Instead, consider examining continuous predictors for nonlinearity using methods such as fractional polynomials or restricted cubic splines, and define categorical predictor groups using a prespecified method. Dichotomising continuous predictors such as age or blood pressure often at arbitrary cut points leads to a loss of information. Selecting cut points through data dredging on the same data set to identify the "optimal" cut point that maximises predictor effects or minimises associated *P*-values, or using a selection procedure to identify "significant thresholds," can substantially reduce the predictive ability of the model. The use of widely predefined cut points (that is, not based on the data at hand) or categorisation into four or more groups carries a lower risk of bias. [TRIPOD-AI [16], STANDING Together 2023 [27]]

[w] All participants enrolled in the study are included in the data analysis. If there are no missing values of predictors or outcomes and the study explicitly reports that participants are not excluded on the basis of missing data, or if missing values are handled using multiple imputation. Both calibration and discrimination are evaluated appropriately (including relevant measures tailored for models predicting survival outcomes). If internal validation techniques, such as bootstrapping and cross-validation including all model development procedures, have been used to account for any optimism in model fitting, and subsequent adjustment of the model performance estimates have been applied. [TRIPOD-AI [16], APPRAISE-AI [17], STANDING Together 2023 [27], FUTURE-AI [47], The medical algorithmic audit [26]]

[x] It is strongly recommended to evaluate and document the expected benefits of the model within its medical context. One approach to achieve this is through decision curve analysis, which assesses the clinical value of a predictive model by weighing the net benefits of using the model

across various decision thresholds, thereby helping to determine its practical impact on decision-making. Additionally, a more robust and comprehensive method to examine the potential impact on medical practice during the early stages of developing an AI-based application is conducting an early Health Technology Assessment. This process systematically evaluates the medical, economic, and social implications of introducing new technologies, providing a thorough understanding of its feasibility and benefits in real-world healthcare settings. [AIPA [24], TRIPOD-AI [16], APPRAISE-AI [17]]

[y] When evaluating potential outcomes, it is recommended to align with the quadruple aim model for value-driven care, which focuses on improving patient experience, reducing care costs, enhancing population health, and improving care provider perception. Potential outcomes include process improvements such as faster diagnoses, earlier hospital discharge, or improved workflows (e.g., home monitoring). Short-term health outcomes may involve immediate health benefits for patients, while long-term outcomes might include enhanced survival, quality of life, or prevention efforts. Additionally, consider societal impacts, such as cost-effectiveness and broader system efficiencies. To ensure a valid assessment of the added benefits of implementing an AI tool in daily medical practice, a comparative study is essential. Ideally, this involves a randomised comparative design with two groups: a control group receiving standard care without the AI application and an intervention group where end-users are informed of and act on the AI recommendations. Alternative designs such as controlled before/after studies, interrupted time series, geographic comparisons, or cross-sectional randomisation (randomisation at the level of a single decision point or event) focused on treatment decisions may be used when randomisation is not feasible. [AIPA [24], ALTAI [13,45], UNESCO Recommendation on the Ethics of Artificial Intelligence 2022 [48], TRIPOD-AI [16]]

[z] To ensure effective implementation of AI tools, continuous monitoring of performance, errors, and fairness is essential. This includes tracking miscalibrations (over- or under-estimation of risk), false positives and negatives (incorrect tumour classifications), and evaluating error margins and data quality over time. Technical errors such as software inaccessibility, integration failures, or slow response times should be addressed promptly through user feedback mechanisms. Fairness must be assessed by analysing outcome disparities across populations particularly for vulnerable groups, using socio-economic health determinants to detect and mitigate bias. [AIPA [24], ALTAI [13,45], UNESCO Recommendation on the Ethics of Artificial Intelligence 2022 [48]]
